# scRNMF: An imputation method for single-cell RNA-seq data by robust and non-negative matrix factorization

**DOI:** 10.1371/journal.pcbi.1012339

**Published:** 2024-08-08

**Authors:** Yuqing Qian, Quan Zou, Mengyuan Zhao, Yi Liu, Fei Guo, Yijie Ding

**Affiliations:** 1 Institute Fundamental and Frontier Sciences, University of Electronic Science and Technology of China, Chengdu, China; 2 Yangtze Delta Region Institute (Quzhou), University of Electronic Science and Technology of China, Quzhou, China; 3 Shenzhen Institutes of Advanced Technology, Chinese Academy of Sciences, Shenzhen, China; 4 School of Computer Science and Engineering, Central South University, Changsha, China; University of California Irvine, UNITED STATES OF AMERICA

## Abstract

Single-cell RNA sequencing (scRNA-seq) has emerged as a powerful tool in genomics research, enabling the analysis of gene expression at the individual cell level. However, scRNA-seq data often suffer from a high rate of dropouts, where certain genes fail to be detected in specific cells due to technical limitations. This missing data can introduce biases and hinder downstream analysis. To overcome this challenge, the development of effective imputation methods has become crucial in the field of scRNA-seq data analysis. Here, we propose an imputation method based on robust and non-negative matrix factorization (scRNMF). Instead of other matrix factorization algorithms, scRNMF integrates two loss functions: *L*_2_ loss and C-loss. The *L*_2_ loss function is highly sensitive to outliers, which can introduce substantial errors. We utilize the C-loss function when dealing with zero values in the raw data. The primary advantage of the C-loss function is that it imposes a smaller punishment for larger errors, which results in more robust factorization when handling outliers. Various datasets of different sizes and zero rates are used to evaluate the performance of scRNMF against other state-of-the-art methods. Our method demonstrates its power and stability as a tool for imputation of scRNA-seq data.

## Author summary

It is still difficult to analyze scRNA-seq data because a significant portion of expressed genes have zeros. Gene expression levels can be restored through the imputation of scRNA-seq data, facilitating downstream analysis. To overcome this challenge, we propose an imputation method based on robust and non-negative matrix factorization (scRNMF). Instead of other matrix factorization algorithms, scRNMF integrates two loss functions: *L*_2_ loss and C-loss. Through the use of several simulated and real datasets, we perform an comprehensively evaluation of scRNMF against existing methods. scRNMF can enhance various aspects of downstream analysis, including gene expression data recovery, cell clustering analysis, gene differential expression analysis, and cellular trajectory reconstruction. The results of our study demonstrate that scRNMF is a powerful tool that can improve the accuracy of single-cell data analysis.

## Introduction

Single-cell RNA sequencing is a powerful technique that allows researchers to analyze gene expression at the single-cell level. However, scRNA-seq data is prone to dropout events, which refer to the failure of detecting true gene expression due to technical limitations during the experimental process. Dropout events occur when the expression level of a gene falls below the detection limit, resulting in zero or low read counts. Dropout events are particularly common in lowly expressed genes, making their identification and handling challenging. Understanding dropout events is crucial for the accurate interpretation of scRNA-seq data.

To address dropout events, several computational methods have been developed. These methods aim to impute the missing gene expression values by leveraging the information from other genes or cells. In general, we can categorize the previous imputation methods into three classes.

The first category of methods involves probabilistic models. These models identify the zeros as dropout values and predict the missing value. scImpute [1] estimates dropout rates using a Gamma-Normal mixture model. For the expression of genes with high dropout probabilities, scImpute constructs a separate non-negative least squares regression model to impute. Huang et al. [2] developed an expression recovery tool called SAVER which uses a Poisson-Gamma model to pool expression values across genes within each cell. Further, SAVER-X, proposed by Wang et al. [3], couples an autoencoder (AE) with a Bayesian model to extract transferable gene-gene relations across data sets.

The second category of methods aims to restore the expression value from the raw data or prior knowledge (such as cell-cell and gene-gene interaction network). For example, MAGIC [4] shares information between similar cells through data diffusion. To capture gene and cell similarities, scTSSR [5] simultaneously learns two non-negative sparse self-representation matrices. Genes and cells that show similarity are bilinearly combined to impute dropout values. Further, scTSSR2 [6] combines matrix decomposition with scTSSR, leading to fast two-side sparse self-representation to impute dropout events in scRNA-seq data. Using a multi-objective optimization model, scMOO [7] infers the combination of weights and the latent representation of three types of structures (horizontal, vertical, and low-rank) from the data.

Another category of methods is based on deep learning (DL) theory. The hidden distribution of gene expression can be captured using a DL-based approach [8–10]. AutoImpute [11] uses an autoencoder (AE) to learn the distribution of input data, imputing the missing values with minimal impact on gene expression levels. The missing values are imputed in DCA [12] by a deep counting AE whose output layer is seen as Zero-inflated Negative Binomial (ZINB) regression. However, these DL-based approaches rely on strong distribution assumptions, which severely limit their effectiveness and utility. Thus, Li et al. [13] developed AutoClass, an algorithm that combines an AE with a classifier without assuming any particular data distribution, allowing it to effectively remove noise and artifacts from scRNA-seq datasets. Wang et al. [14] developed the single-cell Graph Neural Network (scGNN), which uses graph neural networks to learn cell-cell relationships. scIGANs, a proposal by Xu et al. [15], uses generated cells to balance the performance between major and rare cell populations. The scGCL method proposed by Xiong et al. [16], employs a ZINB AE and graph contrastive learning for estimating dropout rates.

MF [17–21] and AE [8, 11–13, 16, 22]-based imputation methods first identify a latent space representation of cells or genes. They then reconstruct the observed expression matrix from the estimated latent spaces, resulting in a matrix that is no longer sparse. While MF is a shallow model capturing the linear relationship between cells and genes, AE is a DL model that can capture non-linear relationships and restore complex structures not exhibited in the raw data. According to comparison results on the single-cell Imputation Methods Comparison platform (scIMC [23]), it is evident that DL-based approaches hold significant potential for imputation. However, AEs often require substantial training data to extract meaningful representations. Insufficient data can lead to overfitting, hindering the model’s ability to generalize accurately to new data.

Research [24, 25] has shown that leveraging bulk RNA-seq data can significantly improve the quality of imputed data. SCRABBLE [26] enhances clustering quality and cell type identification by imputing dropout events from bulk RNA-seq data. It employs matrix regularization rather than relying on cell-cell distance, transforming the mathematical model into a convex optimization problem. DURIAN [27] reduces its error rate by using celltype-specific gene expression patterns in the bulk expression data. During the imputation task, DURIAN enhances the usefulness of bulk data as well as single-cell data by sharing information iteratively. Bubble [28] is an AE-based model that uses matched bulk RNA-seq data to identify and impute scRNA-seq data. With Bubble, the alignment between aggregated imputed data and bulk RNA-seq data improves, resulting in more accurate gene expression level estimation. For imputation, these methods rely on extensive sets of RNA-seq data. However, when bulk RNA-seq data is unavailable, or if there is limited congruence between scRNA-seq and bulk RNA-seq data sets, these methods might become less effective or even unsuitable.

The observed scRNA-seq data can be modeled as a gene count matrix. Matrix factorization (MF), which approximates a data matrix as the product of two or more low-dimensional factor matrices are popular approaches for scRNA-seq data analysis [18, 29]. For example, McImpute [17] uses Nuclear Norm-based MF to recover the full gene expression from partial information. CMF-Impute [20], a collaborative MF method, exploits the information of cell similarity and gene similarity. ALRA adopts low-rank MF to reconstruct scRNA-seq data. ALRA [18] preserve true biological zeros at zero count by set all the values below the threshold to zero. The above MF-based imputation methods utilize *L*_2_ loss in optimization, which can perform well under Gaussian and zero mean noise as assumption [30]. Previous studies [31, 32] show that the *L*_2_ loss measure is sensitive to outliers. In the scRNA-seq data, the raw counts exists false (dropout) zero counts, which are outliers. In this case, the *L*_2_ loss may not properly represent the error statistics and the performance of MF algorithms may degrade.

A large number of imputation methods have been proposed and most of them achieved good performance in different scenarios. However, some benchmark studies [23, 33, 34] find that there still a lack of imputation methods that can perform well across all scenarios. For example, Hou et al. [33] performed a systematic evaluation of 18 scRNA-seq imputation methods. And, they found that the majority of the methods did not improve performance in downstream analyses compared to no imputation, in particular for clustering and trajectory analysis. In Cheng et al.’s study [34], no imputation method performed consistently well across all datasets and some methods even had a negative effect on cell clustering. Furthermore, scIMC [23] designed a comprehensively comparing framework for interpolation methods. The comparing experiment is conducted from the following four aspects: gene expression recovering, cell clustering, gene differential expression and cellular trajectory reconstruction.

**Fig 1:**
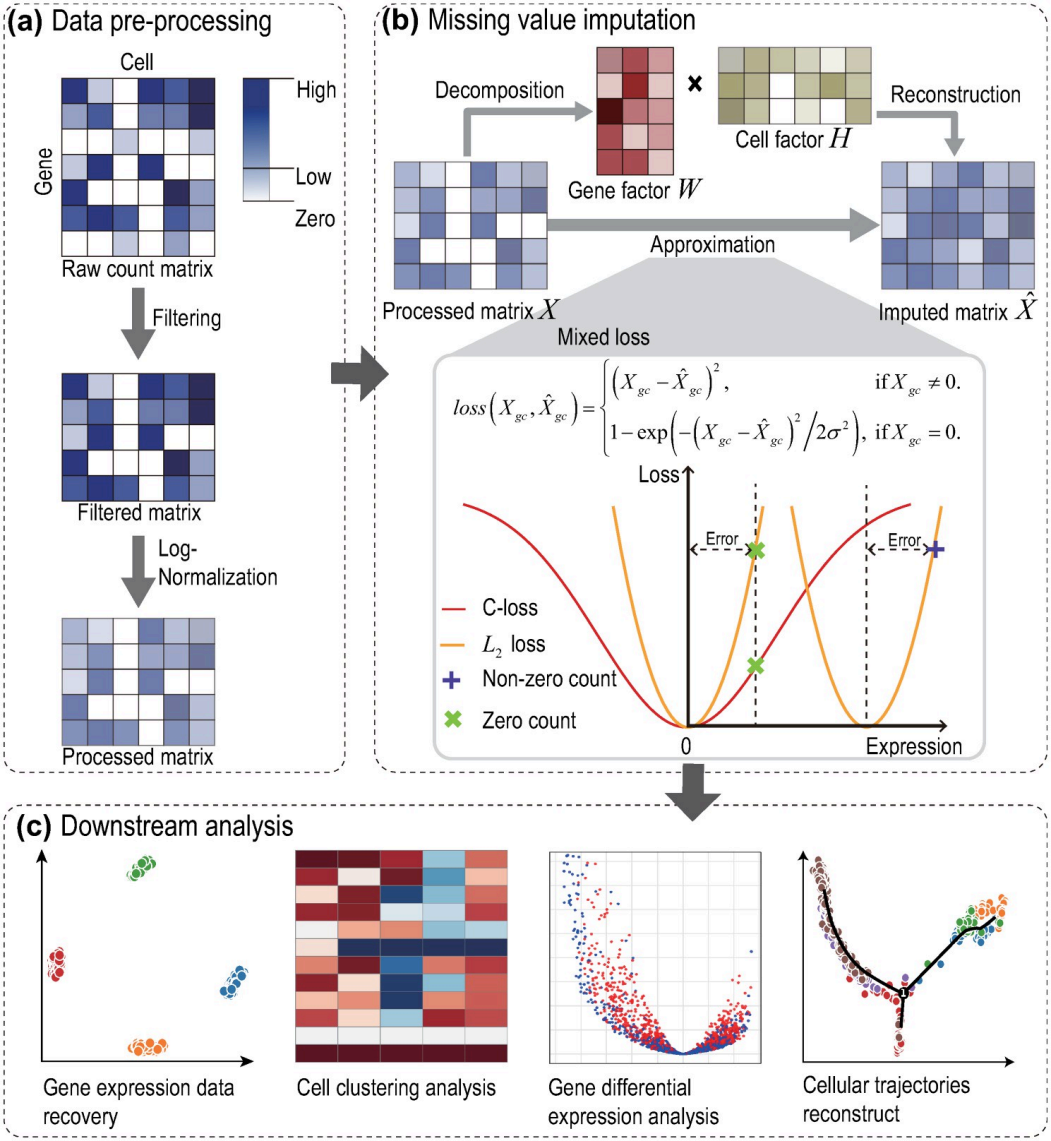
The overview of the scRNMF framework. (**a**) Data pre-processing. Before imputing dropouts, all datasets are filtered out by removing low expression genes. And then, we apply Log-Normalization on the filtered matrix to obtain a processed matrix *X*. (**b**) Missing value imputation. scRNMF is an extension of matrix factorization. scRNMF aims to find two low-rank matrices whose product provides a good approximation to the original matrix. scRNMF integrates two loss function: *L*_2_ loss and C-loss. The *L*_2_ loss function is highly sensitive to outliers, which can introduce substantial errors. We utilized the C-loss function when dealing with zero values in the raw data. (**b**) Downstream analysis. The imputed matrix is used for downstream analysis.

To alleviate the above concerns, we intend to develop a robust imputation method that can handle dropout zeros effectively and improve performance in all scenarios. Here, we extend the typical MF approach and adapt it to solve imputation tasks in scRNA-seq data. To this effect, we develop a MF with a non-negative constraint and C-loss function, termed scRNMF. The contributions of our work are as follows:

(1) Instead of known MF methods [7, 20, 35–37], scRNMF integrates two loss function: *L*_2_ loss and C-loss. Observed zero values in scRNA-seq data don’t necessarily indicate true gene expression, but rather, they signify unobserved values. When we incorporate the C-loss function into our model, a minor penalty is imposed on these zeroes, as illustrated in Fig 1(b). Contrarily, the *L*_2_ loss function assigns a significantly larger penalty for the same error. This distinction leads scRNMF to provide more reliable factorization, particularly in handling outliers.
(2) We develop an iterative algorithm that uses half-quadratic minimization to solve the non-quadratic and non-convex objective function encountered in scRNMF. The objective function is minimized until the convergence of the algorithm is reached. This approach provides an efficient and accurate solution to scRNMF.
(3) Through the use of several simulated and real datasets, we perform an comprehensively evaluation of scRNMF against existing methods. scRNMF can enhance various aspects of downstream analysis, including gene expression data recovery, cell clustering analysis, gene differential expression analysis, and cellular trajectory reconstruction. The results of our study demonstrate that scRNMF is a powerful tool that can improve the accuracy of single-cell data analysis.

## Materials and methods

The overview of our framework is illustrated in Fig 1. It can be seen that the workflow of our work including three steps: data pre-processing, missing value imputation and downstream analysis. The goal of our study is missing value imputation, namely recover the true data from the raw matrix. In this section, we introduce the data preprocessing and imputation method in detail.

### Data pre-processing

Let *X* be the raw count matrix. We index genes and cells using *g* and *c*, respectively. The preprocessing of scRNA-seq data consists of three steps. The first step is data filtering, which removes low-abundance genes and cells, followed by log normalization to account for differences in sequencing depth, and then top gene selection to reduce the dimensionality of the matrix. The log normalization function is defined as:

$$\begin{array}{c} N(X_{gc})=\text{ln}(\text{Median}(X)\frac{X_{gc}}{\sum_{i=1}^{C}X_{gi}}+1) \end{array}$$ (1)

where Median(*X*) is the median of the total cell expression counts. We refer to AutoClass [13], selecting 2000 highly variable genes (HVGs) for imputation.

### Model

The missing value of a cell gene expression count is modelled as a linear combination of gene and cell activation patterns over latent representations. An imputed value is obtained by multiplying each of *k* cell factor components by its corresponding gene factor component, then summing the results. Thus, the missing gene expression count of a gene *g* for a given cell *c* can be expressed as a combination of *k* components as follows:

$$\begin{array}{c} {\hat{X}}_{gc}=\sum_{i=1}^{k}W_{gi}H_{ic} \end{array}$$ (2)

The loss function for MF originally used to measure the quality of approximation was *L*_2_. In many tasks, it has shown its effectiveness due to its mathematical properties. For scRNA-seq data imputation, however, it is not the best choice. The observed zero values do not reflect real gene expression, which can be seen as outlier. The *L*_2_ loss makes MF sensitive to outliers. To address this issue, we propose to replace the quadratic form of residues by the correntropy induced loss (C-loss) function to achieve robust factorization. C-loss function is defined by:

$$\begin{array}{c} l_{C}(y,x)=1-\text{exp}\{-\frac{{\left(y-x\right)}^{2}}{2\sigma^{2}}\} \end{array}$$ (3)

where *σ* is window width. S1 Fig shows the *L*_2_ loss and C-loss function under different widths *σ*. From S1 Fig, we can see that C-loss is a bounded, smooth and non-convex loss, and C-loss behaves like *L*_2_ loss for small errors. S2 Fig displays that, when we use C-loss on zero counts, small punishment can be imposed on the error. This is to say, C-loss is robust to outliers. Since the observed high-expressed gene expressions are usually accurate [20], we use *L*_2_ loss to measure the error.

Thus, we propose the following scRNMF to learn the data latent representation:

$$\begin{array}{c} \begin{array}{cc} & \underset{W,H}{\text{arg}\;\text{min}}\;\frac{1}{2}\sum_{\left\{(g,c)|X_{gc}\neq 0\right\}}{\left(X_{gc}-\sum_{i=1}^{k}W_{gi}H_{ic}\right)}^{2}\\ & \;\;+\frac{1}{2}\sum_{\left\{(g,c)|X_{gc}=0\right\}}l_{C}(X_{gc}-\sum_{i=1}^{k}W_{gi}H_{ic})\\ & \;\;+\frac{\alpha }{2}\|K_{G}-WW^{T}{\|}_{F}^{2}+\frac{\beta }{2}\|K_{C}-H^{T}H{\|}_{F}^{2}\\ & \;\;+\frac{\lambda }{2}\sum_{\left\{(g,c)|X_{gc}\in \{0\}\right\}}{\left(\sum_{i=1}^{k}W_{gi}H_{ic}\right)}^{2}\\ & \text{subject}\;\text{to}:W\geq 0,H\geq 0. \end{array} \end{array}$$ (4)

In cases where the raw data contain a significant amount of redundant information, the cell and gene factor cannot effectively represent cell and gene. To avoid that, the third and fourth terms are introduced. The learned *W* and *H* are consistent with the cell-cell graph *K*_*C*_ and the gene-gene graph *K*_*G*_, respectively. In our study, we take average of the Cosine similarity and Correlation coefficient similarity [38] to describe the distance.

The fifth term is a regularization factor. The goal is to fit the zeros to the fitting constraint. A parameter λ is used to control how important zeros are during learning.

We also introduce a non-negative constraint for factors. It is due to the fact that gene expression counts are not negative. This constraint helps us obtain more accurate results from our model. Moreover, it prevents unrealistic values from being generated by the model. Lastly, it provides a interpretation of the results, as they are constrained to non-negative values.

### Optimization

The objective function as defined in Eq 4 is non-convex, which poses challenges because it cannot be minimized directly. In this context, the half-quadratic optimization algorithm is employed to address this difficulty and optimize function 4. By doing so, the scRNMF issue is broken down into solving the Weighted NMF problem. The optimization procedure consists of two key stages.

The first stage necessitates introducing an additional auxiliary variable. Relying on the principle of the conjugate function [39] and half-quadratic theory [40], the objective function 4 simplifies to Eq 5.

$$\begin{array}{c} \underset{L}{\text{arg}\;\text{min}}\;\sum_{\left\{(g,c)|X_{gc}=0\right\}}(-L_{gc}\frac{{\left(X_{gc}-(WH{)}_{gc}\right)}^{2}}{2\sigma^{2}}+g(L_{gc})) \end{array}$$ (5)

where *g*(⋅) is conjugate function. According to half-quadratic theory [40], the close-form solutions of function 5 is

$$\begin{array}{c} L_{gc}=-\text{exp}\{-\frac{{\left(X_{gc}-(WH{)}_{gc}\right)}^{2}}{2\sigma^{2}}\},(g,c)\in \{(g,c)|X_{gc}=0\} \end{array}$$ (6)

The second stage involves determining *W* and *H* after *L* has been fixed.

$$\begin{array}{c} \begin{array}{cc} & \underset{W,H}{\text{arg}\;\text{min}}\;\frac{1}{2}\|M⊙(X-WH){\|}_{F}^{2}\\ & \;+\frac{\alpha }{2}\|K_{G}-WW^{T}{\|}_{F}^{2}+\frac{\beta }{2}\|K_{C}-H^{T}H{\|}_{F}^{2}\\ & \;+\frac{\lambda }{2}\|P⊙(WH){\|}_{F}^{2}\\ & \text{subject}\;\text{to}:W\geq 0,H\geq 0. \end{array} \end{array}$$ (7)

where ⊙ indicates element-wise matrix multiplication, *P* is weighted matrix and *M* is projection matrix, that is, *P*_*gc*_ = 1 if *X*_*gc*_ ≠ 0 or *P*_*gc*_ = 0 otherwise; *M*_*gc*_ = −*L*_*gc*_ if *X*_*gc*_ = 0 or *M*_*gc*_ = 1 otherwise.

Eq 7 is the Weighted NMF problem. To minimise Eq 7, we use an efficient multiplicative learning algorithm [41]. The multiplicative update rules is given by:

$$\begin{array}{c} \begin{array}{cc} & W\leftarrow W⊙\frac{(M⊙X)H^{T}+\alpha K_{G}W}{(M⊙(WH))H^{T}+\lambda (P⊙(WH))H^{T}+\alpha WW^{T}W}\\ & H\leftarrow H⊙\frac{W^{T}(M⊙X)+\beta HK_{C}}{W^{T}(M⊙(WH))+\lambda W^{T}(P⊙(WH))+\beta HH^{T}H} \end{array} \end{array}$$ (8)

The complete algorithm follows a half-quadratic iterative process that involves alternating between the two stages. The first stage deals with updating *L* using Eq 6, while the second stage updates *W* and *H* according to Eq 8. This iterative process assists in the minimization of the non-convex function until convergence. The details of optimization procedure, the proof of convergence and the pseudo-code for implementation are elaborately defined in S1 Text. We also plot the objective function value on all datasets. The results (S3 Fig) illustrate that scRNMF has good convergence experimentally.

### Parameters selection

There are five parameters in our methods, including *k*, *σ*, *α*, *β* and λ. *k* represents the latent dimensions of the cell and gene. It is chosen from the set {2,10,20,30,40,50}. The window width *σ* is selected from the range 10^−5^ to 10^3^ with intervals of 10. The regularization parameters *α*, *β* and λ are selected from the range 10^−5^ to 10, also with step of 10. We randomly masked 10% of the non-zero counts in the expression matrix and used the RMSE between the imputed values and the masked counts as an evaluation metric for reconstruction error. A low reconstruction error means the model is accurately learning the data and can be used for predictions [12]. We randomly sampled one thousand hyperparameter configurations from the search space. The hyperparameter configuration with the lowest reconstruction error is then selected as the most efficient model. The optimal parameters are listed in S1 Table.

## Results

Multiple datasets and downstream analyses were used to evaluate and compare scRNMF to other imputation methods. The details of datasets and compared imputation methods are list in S2 and S3 Tables.

### Gene expression data recovery

We evaluate expression value recovery on simulated scRNA-seq data. We generate a true counts matrix (matrix without dropouts) and eight additional raw matrix (Simulated 1–8) using Splatter [42] with 500 cells and 1000 genes in four cell groups.

**Fig 2:**
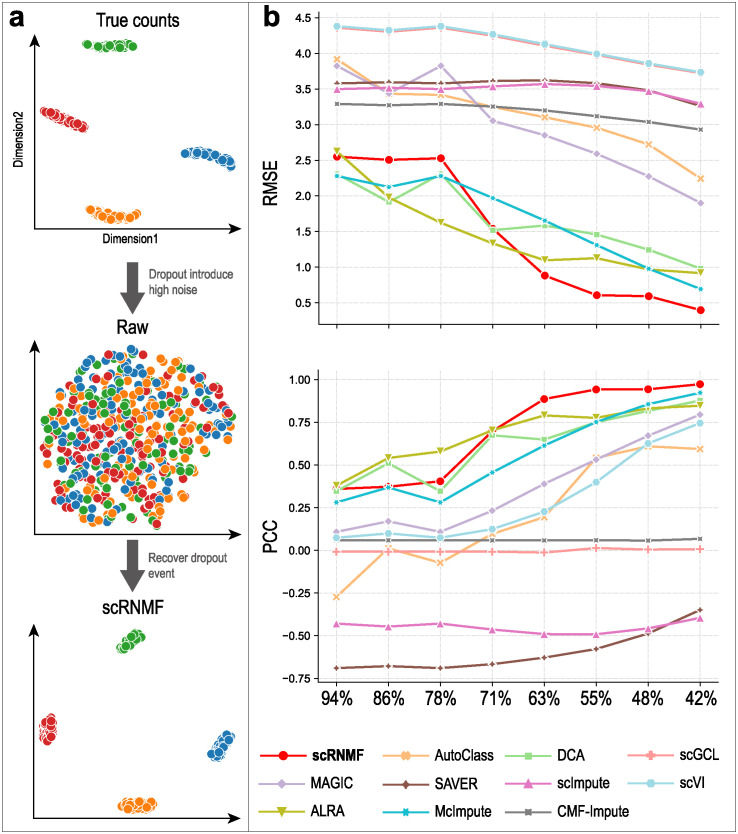
Gene expression data recovery after imputation. (**a**) UMAP plots for true data, raw data and imputed data by scRNMF on the Simulated 1 dataset. (**b**) RMSE and PCC between normalized true counts and imputed values on eight simulated datasets.

The first evaluation method is data visualization. Referring to the study from [13], we use PCA [43] and UMAP [44] for dimension reduction and data visualization. Fig 2a illustrates the results of the Simulated 1 dataset with 78% zero rate. True counts result in four subpopulations with clear borders, whereas raw counts results are affected by dropout noise. After imputation by scRNMF, subpopulations with clear borders were recovered. We also visualize the results of other imputation methods on Simulated 1–6 dataset (which can be found in S4–S9 Figs). On these simulated datasets, we can observe that our method and DCA performed better than other methods, distinguishing four clusters regardless of high or low zero expression rates.

We also evaluate the performance of different imputation methods by using two metrics, Root mean square error (RMSE) and Pearson correlation coefficient (PCC), to quantify their ability to recover true gene expression. Fig 2b shows that as the zero rate increases, the RMSE of all imputation methods increases, while their PCC decreases. A lower RMSE and higher PCC were achieved with scRNMF and DCA, compared with the other methods. CMF-Impute, scGCL and scImpute have the poorest performance, and could not be successfully applied to simulated datasets.

DCA and scRNMF appear to outperform other methods by a significant margin according to visualization and quantitative comparison. In addition, both of them offer a wide range of hyperparameters for tuning the model. AutoClass adds a classifier to the bottleneck layer of the regular autoencoder. Because the default hyperparameters of AutoClass are robust, they work well for the majority of scRNA-Seq datasets. On high zero expression rate simulated data (78%, 71%, 63%), AutoClass does not perform well. This indicates that AutoClass may not be suitable for datasets with a high zero expression rate. Therefore, further optimization of hyperparameters is needed for datasets with a high zero expression rate.

### Cell clustering analysis

In order to identify cell types from scRNA-seq data, clustering is commonly used. A total of five real datasets were used to evaluate the performance of scRNMF for clustering analysis. The size of their datasets ranged from 182 (Buettner) to 8592 (Lake), and the zero rate varied from 38% (Buettner) to 96% (Usoskin).

We implement the cell cluster experiment following AutoClass [13]. Before imputation, we select 2000 HVGs. After imputation, we perform cell clustering using K-means [45] on the imputed and raw datasets. Two metrics were used to evaluate clustering results: Adjusted Rand index (ARI [46]) and Normalized mutual information (NMI). ARI measures the similarity between the clusters and the true classes, while NMI evaluates the mutual information between the two partitions. Both metrics gave high scores, indicating clustering success.

**Fig 3:**
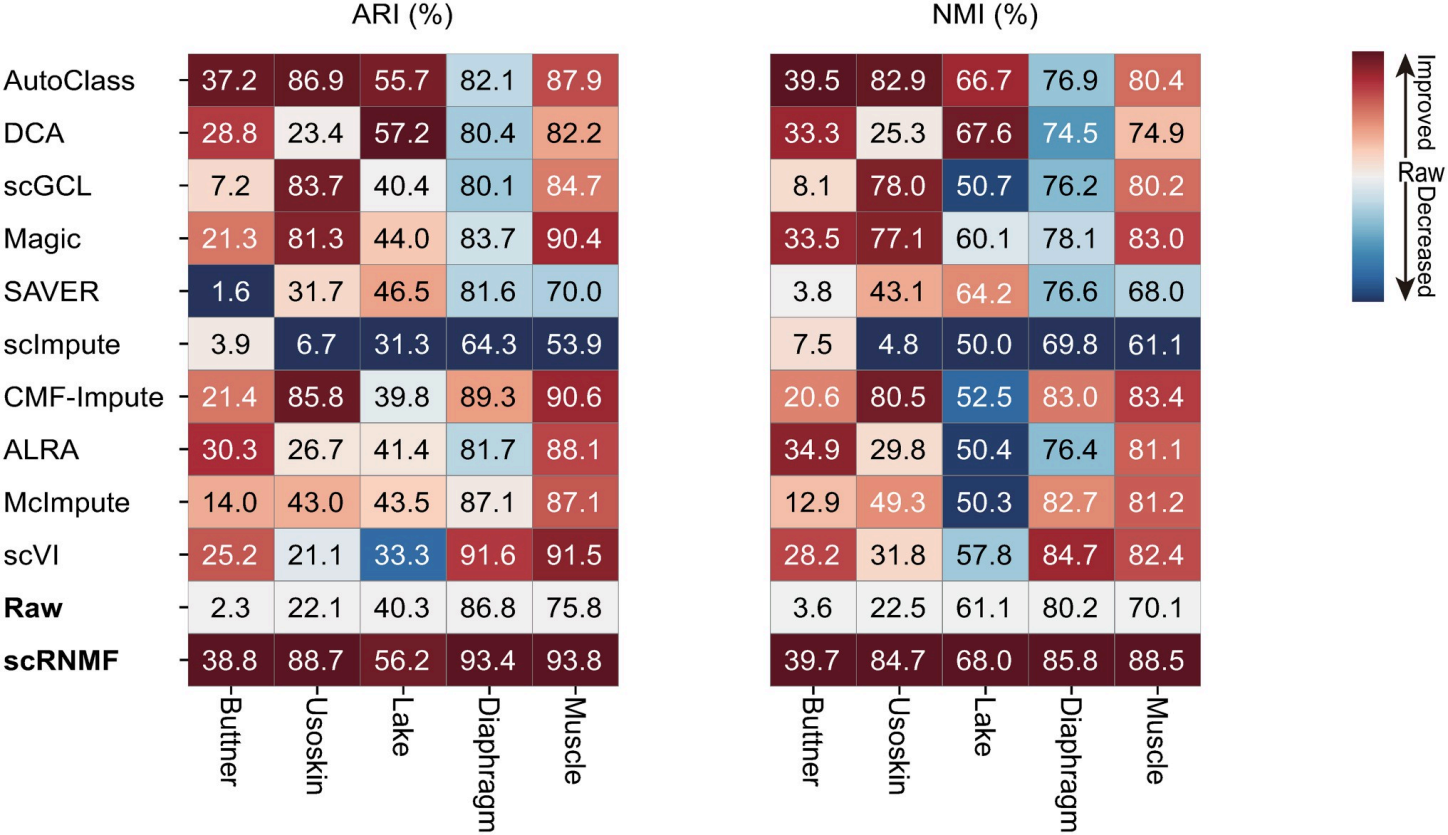
ARI and NMI of cell clustering results of different imputation methods on five datasets.

As indicated in Fig 3, compared with other competing methods, scRNMF achieves the most reliable clustering results in real datasets. Only scRNMF improve two metrics from the raw data in all datasets.

CMF-Impute also achieves competitive performance with ARI reaching 85.8%, 89.3% and 90.6% on Usoskin, Diaphragm and Muscle datasets, respectively. Both scRNMF and CMF-Impute use MF models to impute the raw dataset. The critical difference between the two methods lies in the loss function and the factor constraint. Specifically, CMF-Impute only leverages *L*_2_ loss function to measure the quality of approximation, and has no constraint for factors. While, scRNMF use *L*_2_ loss and C-loss to fit the non-zero and zero value in raw counts, respectively. And, scRNMF introduce non-negative as constraint for factors. Obviously, scRNMF perform better than CMF-Impute in clustering analysis. The behind reason is the raw data contain high noise and gene expression count is non-negative, while CMF-Impute lacks robustness and non-negative constraint.

### Gene differential expression analysis

Further downstream analysis of scRNA-seq data involves identifying differentially expressed (DE) genes. Through this approach, we can identify genes that are specifically associated with the observed phenotypes and gain insight into the molecular mechanisms involved in the process.

**Fig 4:**
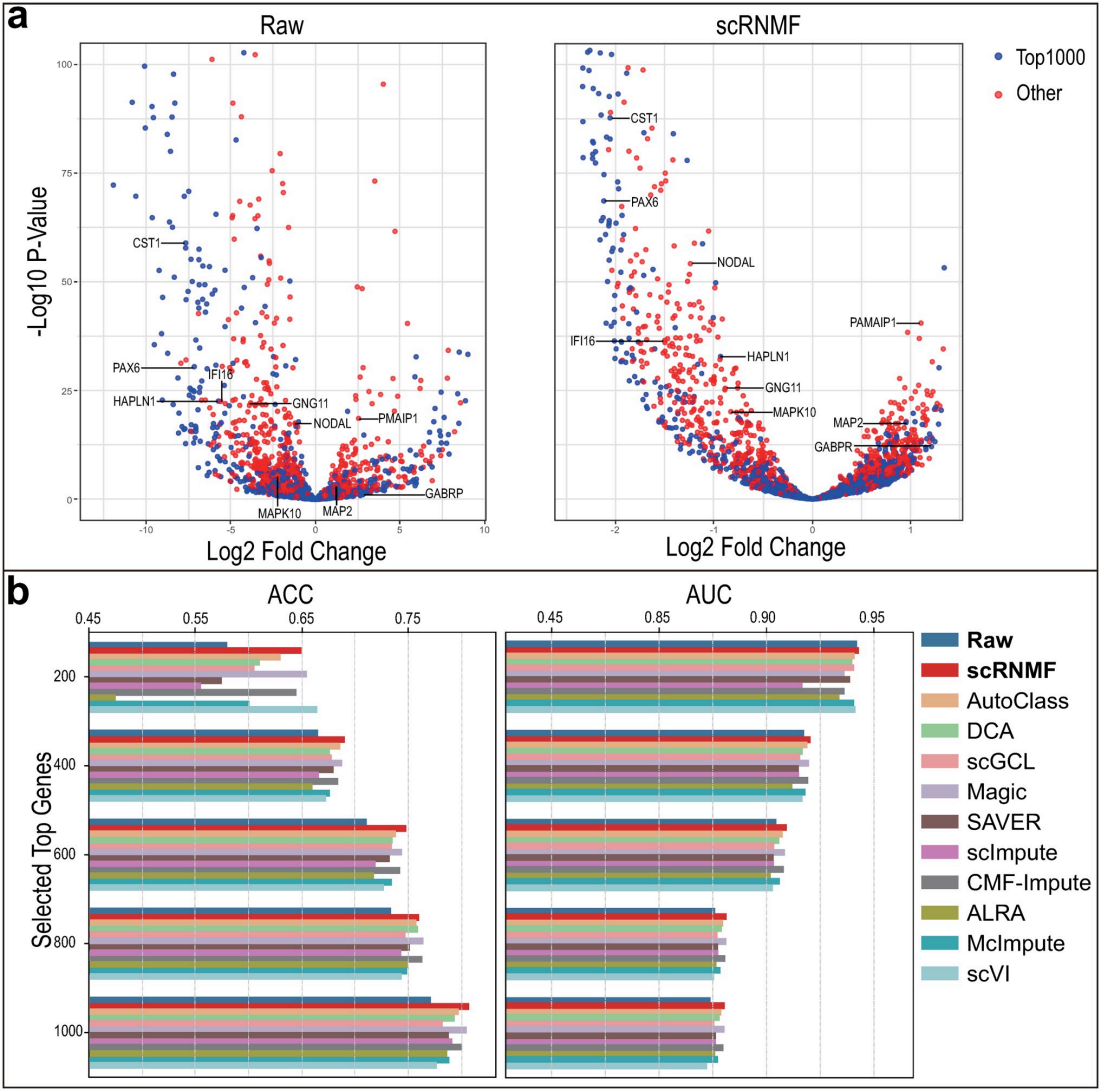
Evaluation of imputation methods through differential expression analysis on H1-DEC dataset. (**a**) Volcano plots of DE genes detected by raw data and imputed data by scRNMF. (**b**) ACC and AUC scores of which the reference are set as the top 200, 400, 600, 800 and 1000 genes sorted by adjusted *P* values from the bulk data.

We implement the DE analysis experiment following scMOO [7]. As bulk RNA-seq data is minimally affected by dropout events, we regarded its differential expression analysis results as our gold standard. We utilized the Cell Type dataset, which incorporates both scRNA-seq and bulk RNA-seq data with seven cell types (H1, DEC, EC, H9, HFF, NPC, and TB). Our primary focus was on detecting DE genes between six pairs of cell subpopulations containing H1. We run edgeR [47] on the raw and imputed data. A top 200, 400, 600, 800 and 1000 gene set based on an adjusted *P* is used as a reference, whereas imputed data is used as a predicted result. Different imputation methods are evaluated based on their Area under the receiver operating characteristic curve (AUC) and Accuracy (ACC). Fig 4, S10, S11, S12, S13 and S14 Figs show the results.

When the top 200 genes are selected, all results shows that only scRNMF outperforms the raw dataset in terms of ACC and AUC. Other imputation methods gradually outperform raw datasets as the number of top genes selected increases. MAGIC and CMF-Impute and also perform well in most cases. We note that these methods all rely on gene similarity. This suggests that using gene similarity for imputation is an effective strategy for better performance, especially in DE analysis.

Besides, we visualize the raw and imputed data with volcano figures as illustrated in Fig 4a. We can see that imputed data from scRNMF detected more accurately the top expressed genes compared with the raw data. Research by Chu et al. [48] shows that DEG cells are enriched in genes such as CST1, PAX6, NODAL and IFI16. Our method have the higher -log(*P*) value in these genes compared with the raw data.

### Cellular trajectories reconstruct

**Fig 5:**
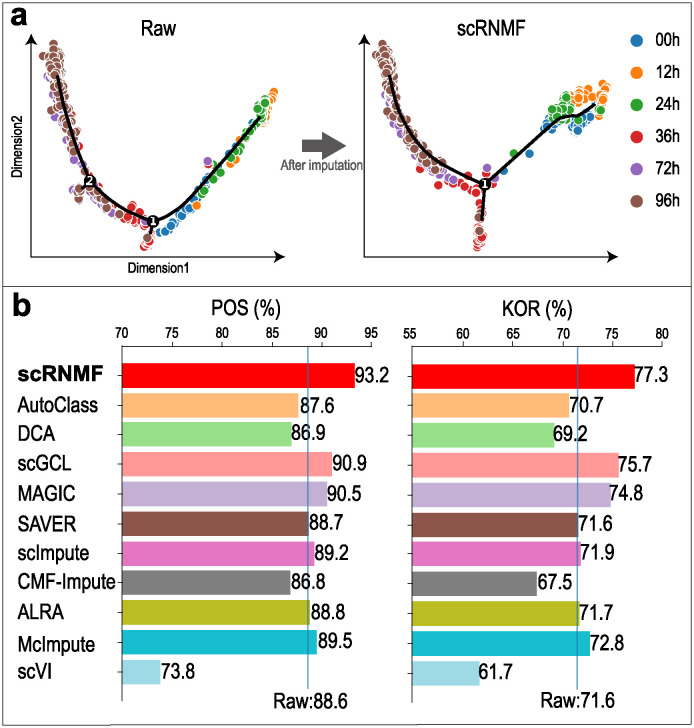
Evaluation of imputation methods through pseudo-time analysis by Monocle 2 on Time-course dataset. (**a**) Visualization of cellular trajectories reconstruction from raw and imputed data. (**b**) POS and KOR scores are used to measure the correlation between the real time labels and the pseudo-time labels.

Cell cycle dynamics patterns can be explored by time course by reconstructing cellular trajectories from scRNA-seq data. Dropout events can lead to incorrect estimates of gene expression levels, which can in turn result in incorrect estimates of cell cycle progression. Therefore, it is critical to take into account the potential for dropouts when reconstructing cellular trajectories in order to ensure that the results are accurate and reliable. In this study, we visualized cellular trajectories reconstructed by Monocle2 [49] (Fig 5(a)). Pseudo-temporal ordering score (POS) and Kendall’s rank correlation score (KOR) [50] scores are used to measure the correlation between the real time labels and the pseudo-time labels (Fig 5(b)). Visualization of cellular trajectories reconstruction from other imputed data on Time-course and Deng datasets are illustrated in S15 and S16 Figs, respectively.

On Deng and Time-course datasets, scRNMF achieves the highest correspondence between imputed data and true cell order (Deng: POS = 94.9%, KOR = 81.1%; Time-course: POS = 93.2%, KOR = 77.3%). Moreover, AutoClass, DCA, CMF-Impute and scVI do not outperform Raw on all datasets. The results suggest that scRNMF and MAGIC are most appropriate for exploring the cellular trajectory in scRNA-seq data.

### Assessing imputation-induced false signals

The observed zero values in scRNA-seq data are not always dropout zeros. There exist true zero events, representing low-level gene expression in a specific cell type [1, 18, 23]. The false positive signals are caused by imputing these true zero events, also known as “over-imputation”.

To evaluate whether different imputation methods lead to false positive signals, we conduct an experiment on simulated scRNA-seq data (because simulated data has true counts matrix as labels). The true zero rate of all simulated datasets are 25.9%. Referring to the experiment from [18, 51], we first define the threshold for binarizing imputed counts. Specifically, the threshold was set based on the percentile of the imputed counts. Counts above this threshold are considered as non-zero, and values below or equal to this threshold are considered as zero. An ideal imputation method should accurately impute the data, in particular preserving at true zero event while completing the dropout ones. Therefore, two metrics were used to evaluate the false positive signals: False Positive Rate (FPR) and F-score. A higher FPR indicates that the imputation method tends to introduce more false positive signals. The F-Score provides a comprehensive evaluation of the method’s performance. A high F-Score indicates that the method achieves a good balance between accurately imputing missing values and not over-imputing true zeros.

**Fig 6:**
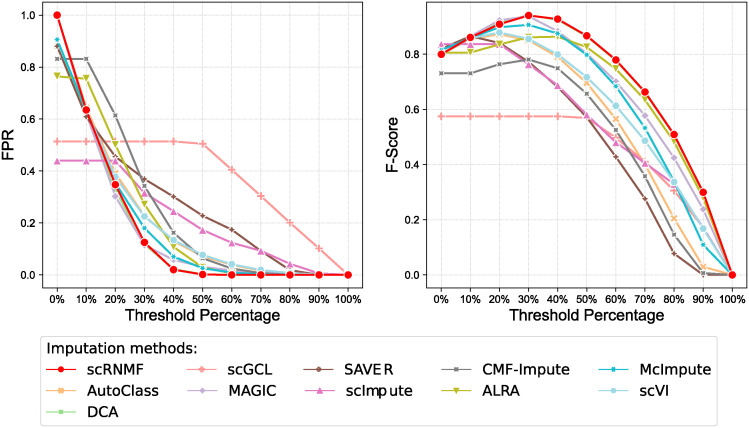
Comparison of imputation methods in reducing false positive signals on Simulated 1 dataset.

We implement experiment about the false positive signals on Simulated 1–6 dataset and the results are show in Fig 6 and S17–S21 Figs. From these figures, we can see that scRNMF, MAGIC and DCA achieve the best F-Score at the 30th percentile on Simulated 1–3 datasets and at the 40th percentile on Simulated 4–6 datasets. At the same time, scRNMF, MAGIC and DCA have the lowest FPR compared to other methods when achieving the best F-score. This indicates that their effectiveness in accurately identifying dropout zeros while maintaining a low false positive signals. Another observation is that ALRA, scGCL and scImpute exhibit the low FPR compared to other methods at 0th percentile on all simulated datasets. This can be explained by the distinct approaches these methods use for imputation. ALRA computes a low-rank approximation of the observed matrix and then restoring true zeros through an entry thresholding process. Differently, scImpute focuses on the identification and subsequent imputation of dropout zeros only. The reconstruction loss utilized by scGCL is the negative log-likelihood of ZINB. As a result, the imputation process of scGCL may lean towards preserving zeros to minimize reconstruction loss.

### Evaluation of parameter *k* selection

The idea behind MF-based imputation methods is to assume that the count matrix can be decomposed into a product of two low-rank matrices, where the matrices are the latent representations of cells and genes, respectively. Therefore, the *k* value is the dimensionality of the latent features of the cells and genes. Inappropriate *k* values can lead to sub-optimal performance [52].

**Fig 7:**
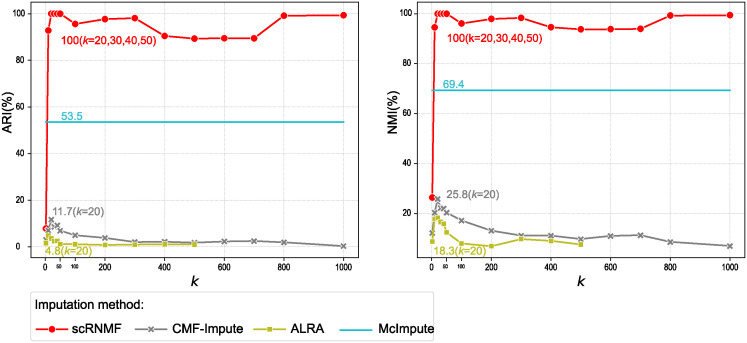
The impact of varying *k* values on the low-rank based imputation methods. Cell clustering analysis is performed on the imputed data, and NMI and ARI are used as evaluation metrics.

To investigate the impact of varying *k* values on the low-rank based imputation methods (including scRNMF, ALRA, CMF-Impute and McImpute), we design simulations using Splatter to generate simulated data. We generate true cell group label and raw matrix (Simulated 9) with 2000 cells, 500 genes and 90% zero rate in 20 cell groups. According to the Louvain algorithm [53, 54], there are 10 gene modules in the Simulation 9 dataset. Cell clustering analysis (the details are the same as in section “Cell clustering analysis”) is performed on the imputed data, and NMI and ARI are used as evaluation metrics. This scenario mimics the biological context where a small number of gene modules produce a variety of cell types through their combinatorial effects. There is a range of values from 2 to 1000 for *k*. This setting helps us understand the effect of increasing *k* due to a growing number of cell type-dependent dropout patterns, even when the true number of genes remains much lower than *k*. The results are shown in Fig 7.

From Fig 7, we can observe that scRNMF achieves the greatest ARI (100%) and NMI (100%) when the *k* value is 20, 30, 40 and 50. As the *k* value increases, ARI and NMI decrease to varying degrees, but when the *k* value reaches 800 and 1000, both NMI and ARI almost reach 100%. This indicates that scRNMF is robust over a wide range of large *k* values. McImpute imposes Nuclear Norm Minimization (NNM) on the imputed count matrix to ensure that it is low-rank. Therefore, McImpute avoid the explicit specification of the *k* value and is a horizontal line in Fig 7. ALRA and CMF-Impute achieve optimal performance when the *k* value is 20. When the *k* value increases, their imputation performance drops significantly. ALRA is based on random SVD [55], so its *k* value cannot be set larger than the minimum number of genes and cells. Another issue worth noting is that ALRA and CMF-Impute chose *k* values of 5 and 40, which are obviously not optimal from the experiment results. ALRA estimates the *k* value from empirical distribution of eigenvalues [56]. CMF-Impute sets the *k* value to the number of cells divided by 50. Our method selects the optimal *k* value by minimizing the reconstruction error, which tends to be accurate but is time-consuming (because running the model multiple times with different parameters).

### Computational time

**Fig 8:**
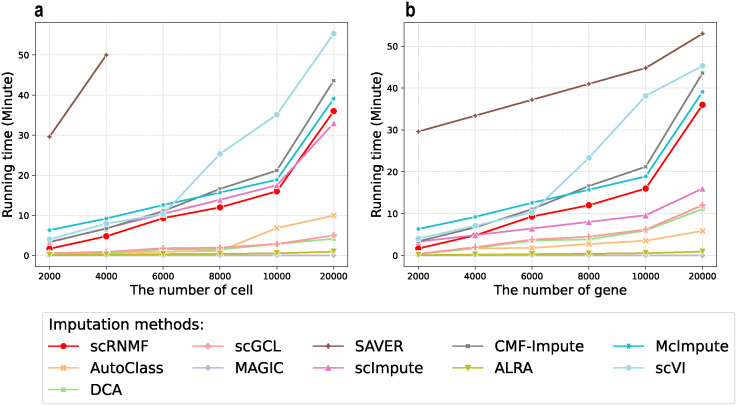
Comparative running time of different imputation methods with a fixed gene count of 2000 (a) and a fixed cell count of 2000 (b).

The computational cost of an imputation method is represented by its running time. The results are depicted in Fig 8, and correspond to computations conducted on a Core i7-10750H CPU with 16GB RAM and an RTX 3060 GPU. Specifically, the GSM4505405 dataset containing 110828 cells and 22966 genes, downloaded from GEO (accession number GSM4505405), was used.

To understand how the cost of different methods changes with the number of cells while keeping the number of genes fixed, we preprocessed the dataset by selecting 2000 HVGs. The dataset was split into six samples with 2000, 4000, 6000, 8000, 10000, and 20000 cells obtained through random sampling. The results are shown in Fig 8(a).

Next, we hold the number of cells constant to witness how the cost of different methods evolved with an increasing number of genes. We selected the top 2000 cells expressing the most genes and filtered out the remaining cells. Then, six datasets featuring 2000, 4000, 6000, 8000, 10000, and 20000 genes were acquired through random sampling. Detailed results are shown in Fig 8(b).

The findings show that scRNMF and the other two MF methods (CMF-Impute and McImpute) exhibit similar running times. And, these times seemed to correlate with the maximum number of cells and genes involved in our experiments. This can be explained by examining the time complexity involved in these methods. Assuming a row count matrix, *X*, sized *M* × *N*, and a known rank, *k* ≪ min(*M*, *N*), let *I* denote the number of iterations needed for the algorithm to converge and *T* represent the maximum of *N* and *M*. For scRNMF, CMF-Impute, and McImpute, the training phase’s time complexity relies heavily on the computation of the matrix product. These methods have a time complexity of *O*(*IT*^2^*k*). Moreover, DL based methods demonstrate a significant speed advantage over MF based methods, particularly with large datasets. This can be attributed to batch gradient descent during the training phase.

Our approach is grounded in MF principles and it might take longer to process extensive datasets. This is an inherent characteristic of matrix operations that we cannot entirely avert. However, this speed trade-off should not overshadow our method’s performance benefits.

## Discussion

It is still difficult to analyze scRNA-seq data because a significant portion of expressed genes have zeros. Gene expression levels can be restored through imputation of scRNA-seq data, facilitating downstream analysis.

In this study, we present a new imputation method, called scRNMF. To measure the quality of approximation, existing MF methods use the *L*_2_ loss function. There is high sensitivity to outliers. When we incorporate the C-loss function into our model, a minor penalty is imposed on these zeroes. Contrarily, the *L*_2_ loss function assigns a significantly larger penalty for the same error. Hence, scRNMF both approximates the original matrix well and improves robustness against dropout events. Due to the non-quadratic and non-convex of scRNMF, we develop an iterative algorithm that relies on half-quadratic minimizations.

To validate the performance of scRNMF, we compare ten imputation methods (S3 Table) and a total of fourteen datasets (S2 Table). For recovering gene expression, we visualize (S4–S9 Figs) the imputed matrix and calculate RMSE and PCC (Fig 2) to evaluate the performance. Compared to other approaches, scRNMF and DCA perform better. In terms of cell clustering, we evaluated K-means on nine real datasets labelled by ARI and NMI scores. As shown in Fig 3, only scRNMF led to improve cell clustering. When evaluating gene DE, EdgeR was run on Cell Type dataset and matched bulk data to calculate ACC and AUC (Fig 4 and S10–S14 Figs). A Moncle2 analysis was carried out on Time-course and Deng datasets (S15 and S16 Figs) in order to investigate the reconstruction of cellular trajectory. There are significant improvements in results for scRNMF over raw dataset.

## Supporting information

S1 Text The details of optimization procedure. Because the objective function of scRNMF is non-convex, we propose an effective optimization algorithm to solve it. We also give the convergence analysis of a optimization algorithm.
(PDF)

S1 Fig C-loss with different widths. C-loss is a bounded, smooth and non-convex loss.
(PDF)

S2 Fig C-loss is robust for zero-count. The observed zero values do not reflect real gene expression, which can be seen as outliers. When we use C-loss on zero count, small punishment (small loss value) can be imposed on the error. In contrast, *L*_2_ loss impose a larger punishment for the same error. This is to say, C-loss is more robust than *L*_2_ loss. Since the observed high-expressed gene expressions (non-zero count) are usually accurate, we use *L*_2_ loss to measure the error.
(PDF)

S3 Fig Convergence curves of the objective function values. We conduct experiments to verify the convergence of scRNMF on all datasets. We plot the objective function value on all datasets. The results illustrate that scRNMF has good convergence experimentally.
(PDF)

S4 Fig PCA+UMAP plots for raw and imputed data on Simulated 1 dataset with zero expression rate of 78%. (PDF)

S5 Fig PCA+UMAP plots for raw and imputed data on Simulated 2 dataset with zero expression rate of 71%. (PDF)

S6 Fig PCA+UMAP plots for raw and imputed data on Simulated 3 dataset with zero expression rate of 63%. (PDF)

S7 Fig PCA+UMAP plots for raw and imputed data on Simulated 4 dataset with zero expression rate of 55%. (PDF)

S8 Fig PCA+UMAP plots for raw and imputed data on Simulated 5 dataset with zero expression rate of 48%. (PDF)

S9 Fig PCA+UMAP plots for raw and imputed data on Simulated 6 dataset with zero expression rate of 42%. (PDF)

S10 Fig Evaluation of imputation methods through differential expression analysis on H1-EC dataset. The ACC (**A**) and AUC (**B**) scores of which the reference are set as the top 200, 400, 600, 800 and 1000 genes sorted by adjusted *P* values from the bulk data.
(PDF)

S11 Fig Evaluation of imputation methods through differential expression analysis on H1-H9 dataset. The ACC (**A**) and AUC (**B**) scores of which the reference are set as the top 200, 400, 600, 800 and 1000 genes sorted by adjusted *P* values from the bulk data.
(PDF)

S12 Fig Evaluation of imputation methods through differential expression analysis on H1-HFF dataset. The ACC (**A**) and AUC (**B**) scores of which the reference are set as the top 200, 400, 600, 800 and 1000 genes sorted by adjusted *P* values from the bulk data.
(PDF)

S13 Fig Evaluation of imputation methods through differential expression analysis on H1-NPC dataset. The ACC (**A**) and AUC (**B**) scores of which the reference are set as the top 200, 400, 600, 800 and 1000 genes sorted by adjusted *P* values from the bulk data.
(PDF)

S14 Fig Evaluation of imputation methods through differential expression analysis on H1-TB dataset. The ACC (**A**) and AUC (**B**) scores of which the reference are set as the top 200, 400, 600, 800 and 1000 genes sorted by adjusted *P* values from the bulk data.
(PDF)

S15 Fig Evaluation of imputation methods through pseudo-time analysis by Monocle 2 on Time-course dataset. (PDF)

S16 Fig Evaluation of imputation methods through pseudo-time analysis by Monocle 2 on Deng dataset. (PDF)

S17 Fig Comparison of imputation methods in reducing false positive signals on Simulated 2 dataset. (PDF)

S18 Fig Comparison of imputation methods in reducing false positive signals on Simulated 3 dataset. (PDF)

S19 Fig Comparison of imputation methods in reducing false positive signals on Simulated 4 dataset. (PDF)

S20 Fig Comparison of imputation methods in reducing false positive signals on Simulated 5 dataset. (PDF)

S21 Fig Comparison of imputation methods in reducing false positive signals on Simulated 6 dataset. (PDF)

S1 Table The parameters of all datasets. (PDF)

S2 Table The details of the scRNA-seq datasets. (PDF)

S3 Table The details of the competing imputation methods. (PDF)
