# Supplementary material for: scRNMF: An imputation method for single-cell RNA-seq data by robust and non-negative matrix factorization
Source: PLoS Comput Biol. 2024 Aug 8;20(8):e1012339. doi: 10.1371/journal.pcbi.1012339 (PMC11338450; doi:10.1371/journal.pcbi.1012339)
Supplement: S1 Text — Because the objective function of scRNMF is non-convex, we propose an effective optimization algorithm to solve it. We also give the convergence analysis of a optimization algorithm. (PDF) [file pcbi.1012339.s001.pdf]

scRNMF: an imputation method for single-cell RNA-seq data by robust and non-negative matrix factorization

YUQING QIAN, YIJIE DING, MENGYUAN ZHAO, YI LIU, FEI GUO AND QUAN ZOU

CONTENTS

1 The proposed model 1

2 Solution to the proposed model 1

3 Initializations of the proposed method 3

4 Stop criteria 3

5 Pseudo-code 3

6 Convergence analysis 4

1. THE PROPOSED MODEL

In our study, we propose a robust and non-negative matrix factorization method, called scRNMF, to impute scRNA-seq data. We learn MF that minimizes the following objective function:

$$\begin{aligned}\arg\min_{W,H} \frac{1}{2} & \sum_{\{(g,c)|X_{gc}\neq 0\}} \left(X_{gc} - \sum_{i=1}^{k} W_{gi}H_{ic}\right)^2 \\ &+ \frac{1}{2} \sum_{\{(g,c)|X_{gc}=0\}} l_C\left(X_{gc} - \sum_{i=1}^{k} W_{gi}H_{ic}\right) \\ &+ \frac{\alpha}{2}\left\|K_G - WW^T\right\|_F^2 + \frac{\beta}{2}\left\|K_C - H^TH\right\|_F^2 \\ &+ \frac{\lambda}{2} \sum_{\{(g,c)|X_{gc}\in\{0\}\}} \left(\sum_{i=1}^{k} W_{gi}H_{ic}\right)^2 \\ \text{subject to} &: W \geq 0, H \geq 0.\end{aligned} \tag{S1}$$

where $\|\cdot\|_F^2$ is the Frobenius norm and l_C is the C-loss function. C-loss function is defined by:

$$l_C(y,x) = 1 - \exp\left\{-\frac{(y-x)^2}{2\sigma^2}\right\} \tag{S2}$$

where σ is window width.

2. SOLUTION TO THE PROPOSED MODEL

Equation S1 is non-convex and non-quadratic. Hence, we develop an efficient iteration method for solving scRNMF.

First, we use the half-quadratic optimization algorithm to rewrite the objective function. A convex function is defined as:

$$g(v) = -v\log(-v) + v, \tag{S3}$$

where $v < 0$. Given the definition of conjugate function [1], we can get the conjugate function $g^*(u)$ of Equation S3 as

$$g^*(u) = \sup_v g'(v), \tag{S4}$$

where

$$g'(v) = uv - g(v) = uv + v\log(-v) - v. \tag{S5}$$

By setting the derivate of $g'(v)$ *w.r.t* to zero, we have

$$v = -\exp(-u) < 0. \tag{S6}$$

Then substituting v with Equation S4, Equation S4 can be written as

$$g^*(u) = \exp(-u). \tag{S7}$$

We can obtain

$$g^*\left(\frac{e_{gc}^2}{2\sigma^2}\right) = \sup\left(\frac{e_{gc}^2}{2\sigma^2}v + v\log(-v) - v\right) = \exp\left\{-\frac{e_{gc}^2}{2\sigma^2}\right\}, \tag{S8}$$

where

$$e_{gc} = X_{gc} - \sum_{i=1}^{k} W_{gi}H_{ic}, \tag{S9}$$

and the supremum is achieved at

$$v = -\exp\left(\frac{e_{gc}^2}{2\sigma^2}\right) < 0. \tag{S10}$$

So far, Equation S1 can be rewrite as

$$\begin{aligned}
&\arg\min_{W,H} \frac{1}{2}\sum_{\{(g,c)|X_{gc}\neq 0\}} e_{gc}^2 + \frac{1}{2}\sum_{\{(g,c)|X_{gc}=0\}}\left(1 - \sup_{v_{gc}}\left(\exp\left(-\frac{e_{gc}^2}{2\sigma^2}\right)v_{gc} - g(v_{gc})\right)\right) \\
&\quad + \frac{\alpha}{2}\left\|K_G - WW^T\right\|_F^2 + \frac{\beta}{2}\left\|K_C - H^TH\right\|_F^2 \\
&\quad + \frac{\lambda}{2}\sum_{\{(g,c)|X_{gc}\in\{0\}\}}\left(\sum_{i=1}^{k} W_{gi}H_{ic}\right)^2
\end{aligned} \tag{S11}$$

subject to :$W \geq 0, H \geq 0$.

In this case, an alternating optimization method is used because solving Equation S11 directly is difficult. The next step involves two steps.

- The first step.

  Given W and H, then Equation S11 becomes

$$\arg\min_v \sum_{\{(g,c)|X_{gc}=0\}}\left(-\frac{e_{gc}^2}{2\sigma^2}v_{gc} + g(v_{gc})\right). \tag{S12}$$

  According to Equation S5, we can easily get the solution

$$v_{gc} = -\exp\left(-\frac{e_{gc}^2}{2\sigma^2}\right) < 0. \tag{S13}$$

- The second step.

Fixing v, Equation (11) can rewrite as:

$$\begin{aligned}\arg\min_{W,H} \frac{1}{2} \sum_{\{(g,c)|X_{gc}\neq 0\}} e_{gc}^2 + \frac{1}{2} \sum_{\{(g,c)|X_{gc}=0\}} \left(-\frac{e_{gc}^2}{2\sigma^2} v_{gc} \right) \\ + \frac{\alpha}{2} \left\| K_G - WW^T \right\|_F^2 + \frac{\beta}{2} \left\| K_C - H^T H \right\|_F^2 \\ + \frac{\lambda}{2} \sum_{\{(g,c)|X_{gc}\in\{0\}\}} \left(\sum_{i=1}^{k} W_{gi} H_{ic} \right)^2 \\ \text{subject to} : W \geq 0, H \geq 0. \end{aligned} \tag{S14}$$

Equation S14 can further rewrite as:

$$\begin{aligned}\arg\min_{W} f(W,H) = \frac{1}{2} \| M \odot (X - WH) \|_F^2 \\ + \frac{\alpha}{2} \left\| K_G - WW^T \right\|_F^2 + \frac{\beta}{2} \left\| K_C - H^T H \right\|_F^2 \\ + \frac{\lambda}{2} \| P \odot (WH) \|_F^2 \\ \text{subject to} : W \geq 0. \end{aligned} \tag{S15}$$

where M is weighted matrix

$$M_{gc} = \begin{cases} 1, if X_{gc} \neq 0 \\ -v_{gc}, if X_{gc} = 0 \end{cases} . \tag{S16}$$

P is projection matrix

$$P_{gc} = \begin{cases} 0, if X_{gc} \neq 0 \\ 1, if X_{gc} = 0 \end{cases} , \tag{S17}$$

and $\odot$ indicates element-wise matrix multiplication.

Obviously, when v is given, the optimization in Equation S11 is reduced to the weighted NMF problem in Equation S15. Therefore, the multiplicative update rules [2] can be directly applied to optimize Equation S15. If we repeat the Lagrange multiplier on the Equation S15 and use the non-negative part of the Karush-Khun-Tucker condition, we obtain the following updating rules:

$$W = W \odot \frac{(M \odot X) H^T + \alpha K_G W}{(M \odot (WH)) H^T + \lambda (P \odot (WH)) H^T + \alpha WW^T W}, \tag{S18}$$

$$H = H \odot \frac{W^T (M \odot X) + \beta H K_C}{W^T (M \odot (WH)) + \lambda W^T (P \odot (WH)) + \beta H H^T H}. \tag{S19}$$

The complete algorithm to solve the objective function S1 is summarized in Supplementary section 5. In Supplementary section 6, we prove that the objective value of Equation S1 is nonincreasing under the iterative algorithm.

3. INITIALIZATIONS OF THE PROPOSED METHOD

Like other NMF algorithms, the proposed objective function S1 is also guaranteed to converge, but may not lead to a global optimum. Therefore, it is dependent on the initial values of W and H. In our method, we employ NNDSVD [3] to initial W and H. NNDSVD is well suited to initialize NMF with sparse factor.

4. STOP CRITERIA

Our algorithm stops when it detects a relative change in W and H that does not exceed the maximum tolerance. The default value of maximum tolerance is 0.01.

5. PSEUDO-CODE

Algorithm S1. An iterative algorithm for the scRNMF model

Input: Normalized expression matrix X; Parameters k, σ, α, β, λ.
Output: Imputed expression matrix $\hat{X}$;
1: Calculate the cell similarity matrix K_C;
2: Calculate the gene similarity matrix K_G;
3: **while** not converged **do**
4: Update v using Equation S13;
5: Update W using Equation S18;
6: Update H using Equation S19;
7: **end while**
8: **Return** $\hat{X} = WH$

6. CONVERGENCE ANALYSIS

In this section, we give convergence proof to show that the values of function S1 is nonincreasing under the update step in Equations S13, S18 and S19.

Theorem: The values of function of S1 monotonically decrease in each iteration until convergence.

Proof: Based on the conjugate function theory [1], we can rewrite the objective function S1 as Equation S11. Thus, we need to prove that the values of function S11 is nonincreasing under the update rule.

Suppose $J\left(W^t, H^t, v^t\right)$ is the value of the function S11 in the tth iteration. In the $(t+1)$th iteration, W^t and H^t are fixed, and the function S11 be written as the subproblem S12. Since the function S12 is convex, then

$$J\left(W^t, H^t, v^{t+1}\right) \leq J\left(W^t, H^t, v^t\right). \tag{S20}$$

When fixing v^{t+1}, Equation S11 is equivalent to $f(W, H)$ (the Equation S15). Next, we need prove that $f(W, H)$ is nonincreasing under the updating step in the Equation S18 and S19. From the theory of constrained optimization [4], we know that to prove it, we need to show that at convergence, the solution satisfies the KKT complementary conditions:

$$W_{gi}\left(\frac{\partial f}{\partial W}\right)_{gi} = 0, \tag{S21}$$

$$H_{ic}\left(\frac{\partial f}{\partial H}\right)_{ic} = 0. \tag{S22}$$

By setting the derivative of f with respect to W and H to zero respectively, we have:

$$\frac{\partial f}{\partial W} = -\left(M \odot (X - WH)\right) H^T + \lambda \left(P \odot (WH)\right) H^T + \alpha\left(K_G - WW^T\right)(-W), \tag{S23}$$

$$\frac{\partial f}{\partial H} = -W^T\left(M \odot (X - WH)\right) + \lambda W^T\left(P \odot (WH)\right) + \beta(-H)\left(K_C - H^T H\right). \tag{S24}$$

At a local minimum, W and H equal to the solution where satisfy the KKT conditions in Equation S21 and S22. Then, substituting Equation S23 with Equation S21:

$$\begin{aligned} &\left(\left(M \odot (WH)\right) H^T\right) \odot W + \lambda\left(P \odot (WH)\right) H^T \odot W \\ &= \left(\left(M \odot X\right) H^T\right) \odot W + \alpha\left(\left(K_G - WW^T\right) W\right) \odot W. \end{aligned} \tag{S25}$$

Substituting Equation S24 with Equation S22:

$$\begin{aligned} &\left(W^T\left(M \odot (WH)\right)\right) \odot H + \lambda W^T\left(P \odot (WH)\right) \odot H \\ &= \left(W^T\right)\left(M \odot X\right) \odot H + \beta H\left(K_C - H^T H\right) \odot H. \end{aligned} \tag{S26}$$

We can rewrite Equation S25 and S26 as:

$$W = W \odot \frac{\left(M \odot X\right) H^T + \alpha K_G W}{\left(M \odot (WH)\right) H^T + \lambda\left(P \odot (WH)\right) H^T + \alpha W W^T W}, \tag{S27}$$

and

$$H = H \odot \frac{W^T\left(M \odot X\right) + \beta H K_C}{W^T\left(M \odot (WH)\right) + \lambda W^T\left(P \odot (WH)\right) + \beta H H^T H}, \tag{S28}$$

respectively.

Therefore, the update step S27 and S28 satisfies KKT conditions and converges to a local minimum. So, we have

$$J\left(W^{t+1}, H^{t+1}, v^{t+1}\right) \le J\left(W^{t}, H^{t}, v^{t+1}\right) \tag{S29}$$

Finally, we combine the above results and obtain the following:

$$J\left(W^{t+1}, H^{t+1}, v^{t+1}\right) \le J\left(W^{t}, H^{t}, v^{t}\right) \tag{S30}$$

REFERENCES

1. S. P. Boyd and L. Vandenberghe, *Convex optimization* (Cambridge university press, 2004).
2. D. Lee and H. S. Seung, “Algorithms for non-negative matrix factorization,” Adv. neural information processing systems **13** (2000).
3. C. Boutsidis and E. Gallopoulos, “Svd based initialization: A head start for nonnegative matrix factorization,” Pattern recognition **41**, 1350–1362 (2008).
4. T. Li and C. Ding, “The relationships among various nonnegative matrix factorization methods for clustering,” in *Sixth International Conference on Data Mining (ICDM’06),* (IEEE, 2006), pp. 362–371.
