# Supplementary figures and images for: scRNMF: An imputation method for single-cell RNA-seq data by robust and non-negative matrix factorization
Source: PLoS Comput Biol. 2024 Aug 8;20(8):e1012339. doi: 10.1371/journal.pcbi.1012339 (PMC11338450; doi:10.1371/journal.pcbi.1012339)

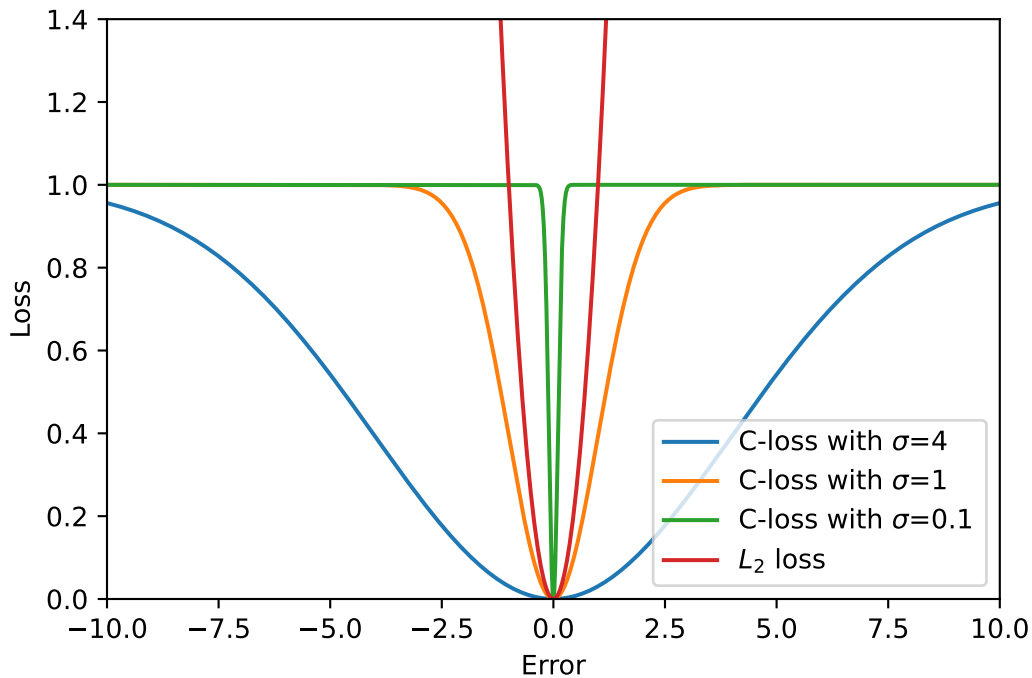

Supplement: S1 Fig — C-loss is a bounded, smooth and non-convex loss. (PDF) [file pcbi.1012339.s002.pdf]

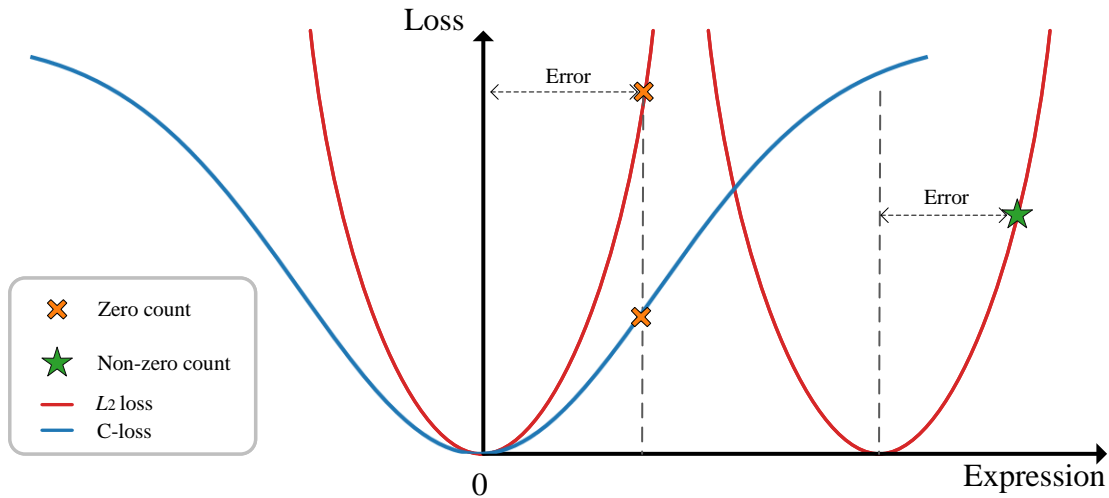

Supplement: S2 Fig — The observed zero values do not reflect real gene expression, which can be seen as outliers. When we use C-loss on zero count, small punishment (small loss value) can be imposed on the error. In contrast, L2 loss impose a larger punishment for the same error. This is to say, C-loss is more robust than L2 loss. Since the observed high-expressed gene expressions (non-zero count) are usually accurate, we use L2 loss to measure the error. (PDF) [file pcbi.1012339.s003.pdf]

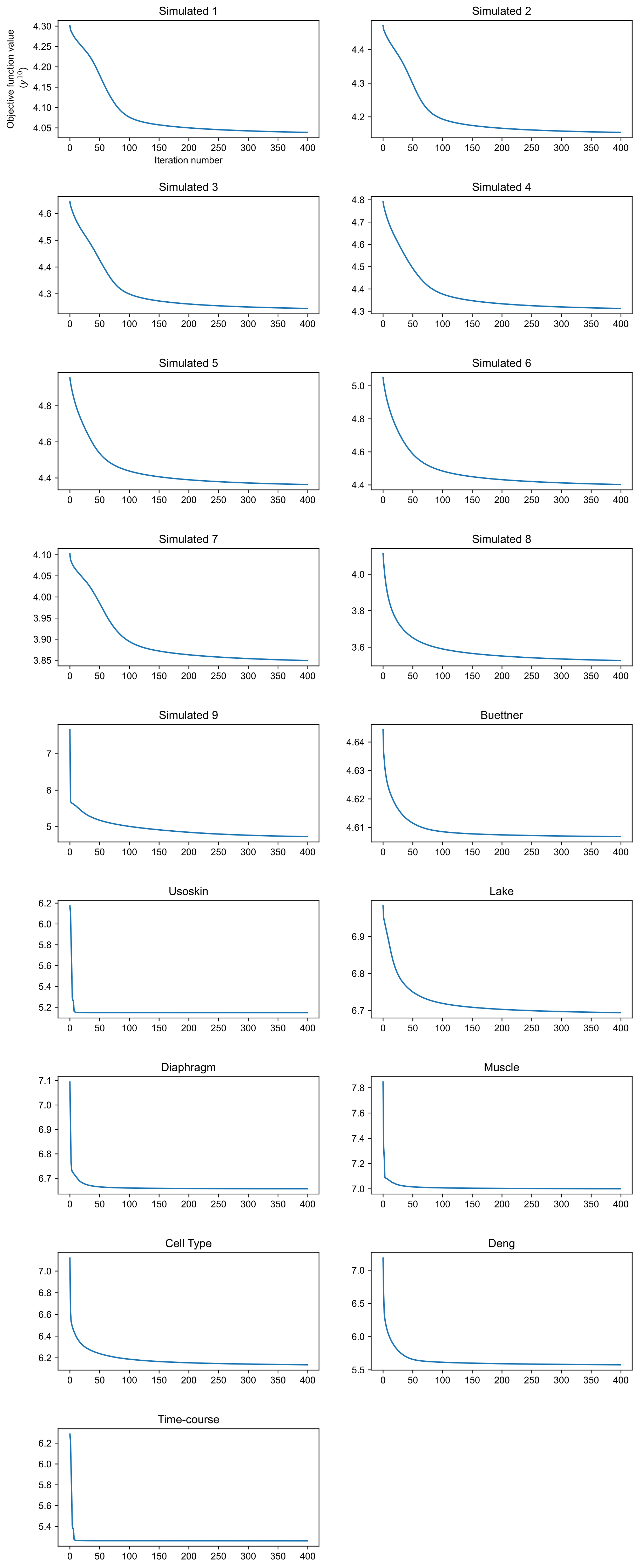

Supplement: S3 Fig — We conduct experiments to verify the convergence of scRNMF on all datasets. We plot the objective function value on all datasets. The results illustrate that scRNMF has good convergence experimentally. (PDF) [file pcbi.1012339.s004.pdf]

A

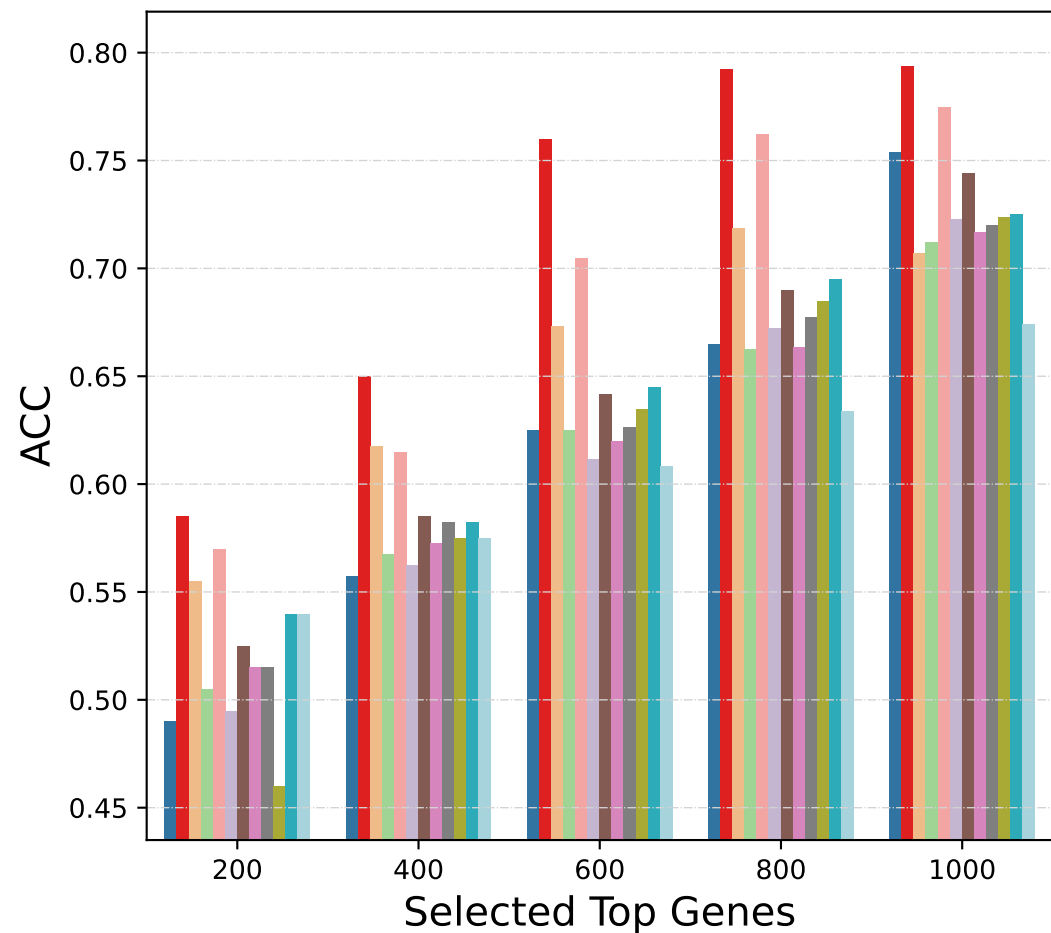

B

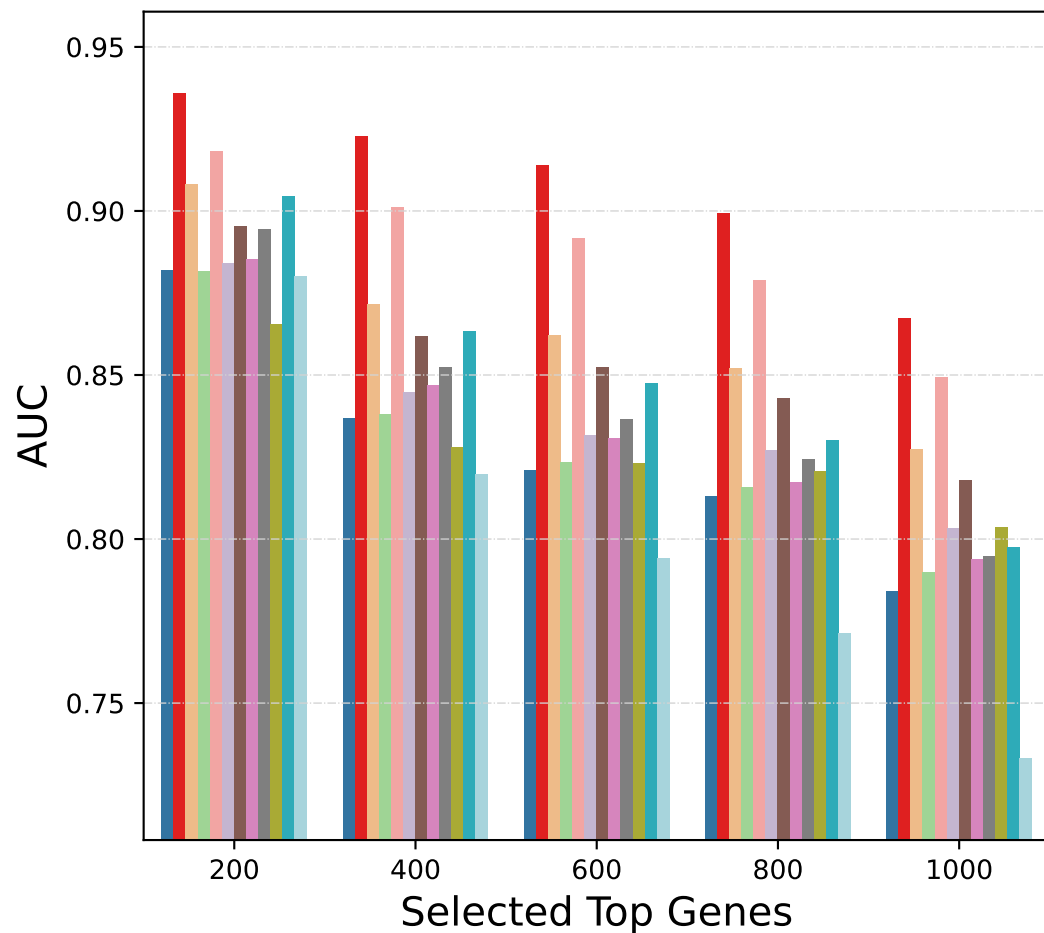

Imputation methods

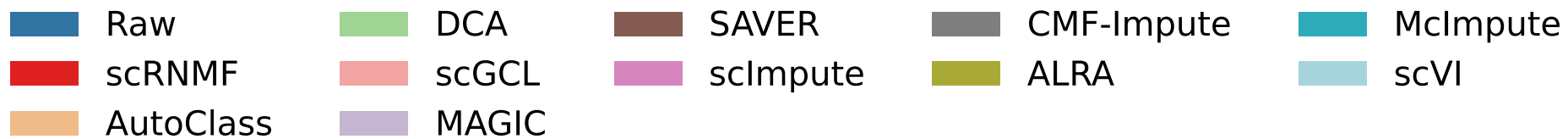

Supplement: S10 Fig — The ACC (A) and AUC (B) scores of which the reference are set as the top 200, 400, 600, 800 and 1000 genes sorted by adjusted P values from the bulk data. (PDF) [file pcbi.1012339.s011.pdf]

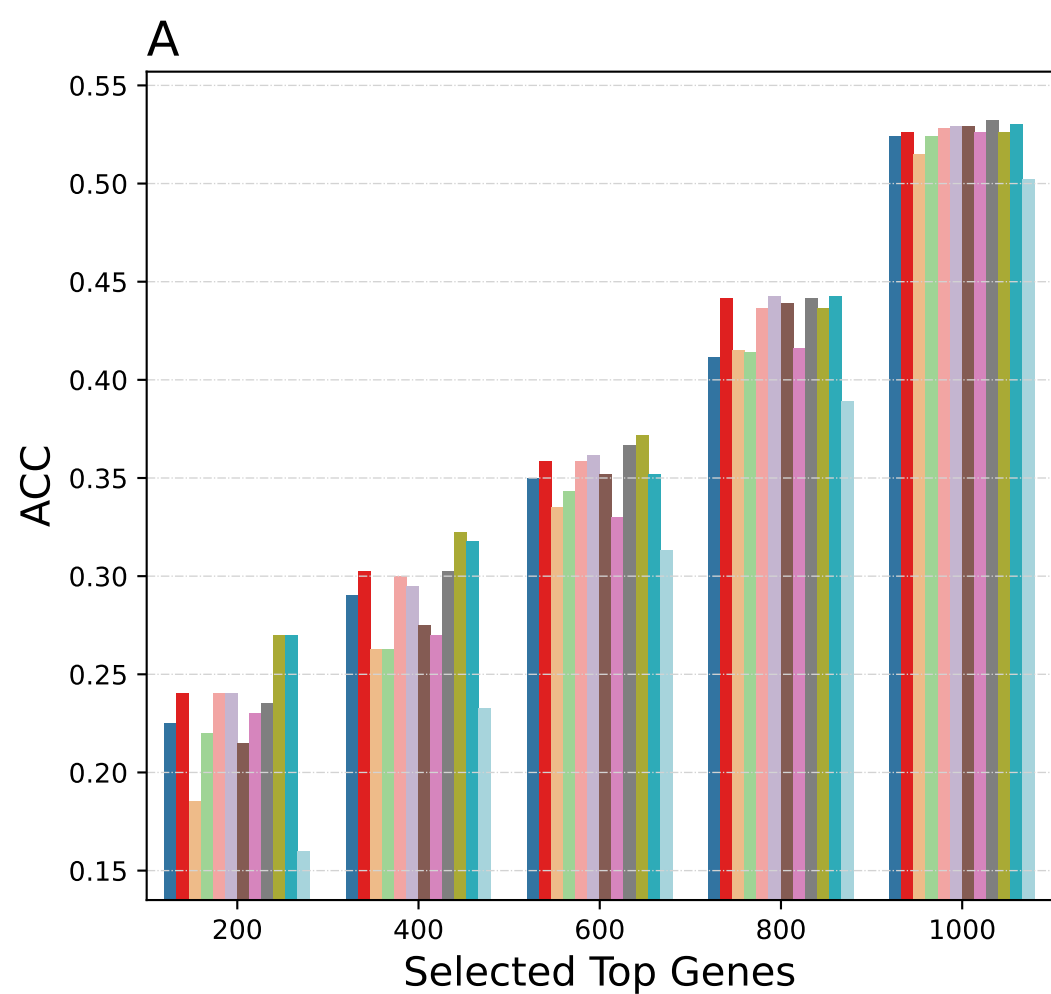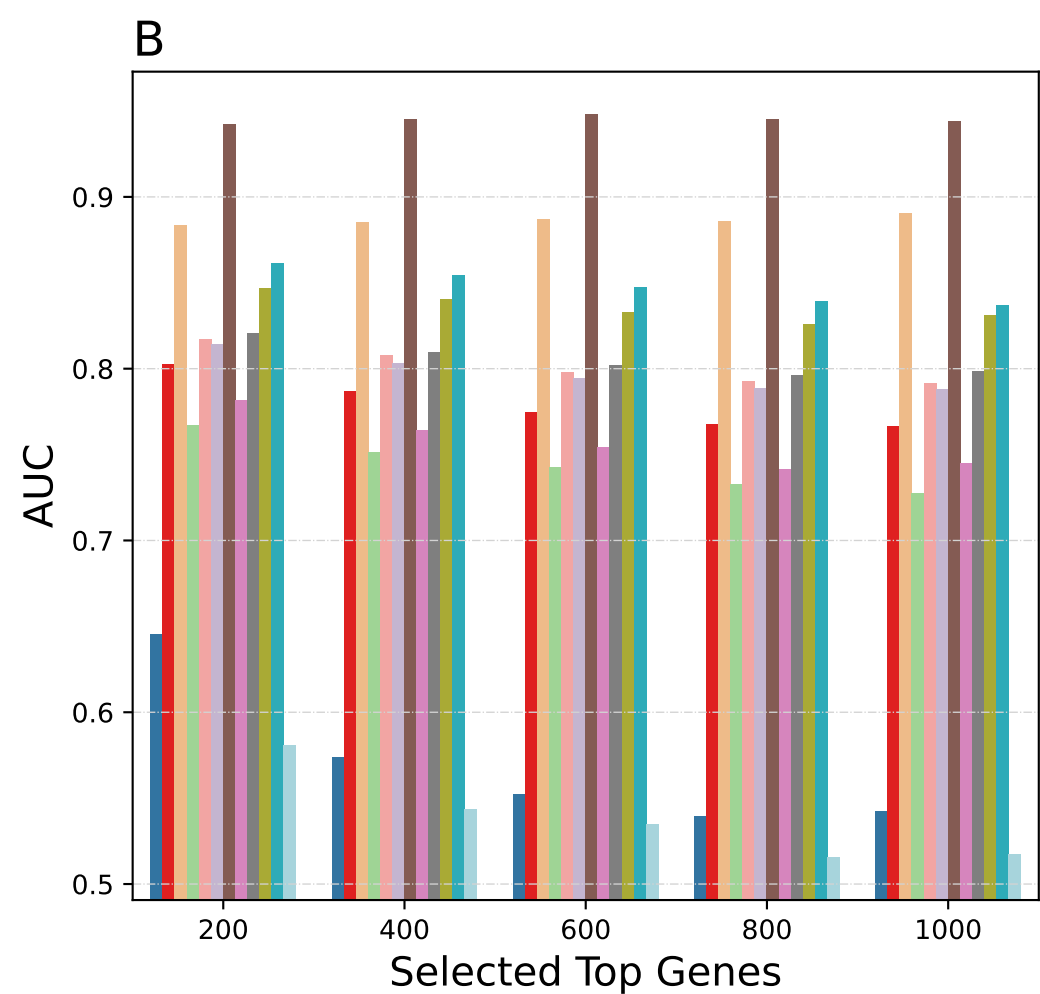

Imputation methods

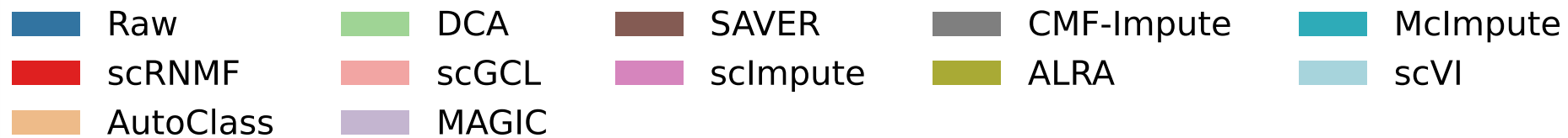

Supplement: S11 Fig — The ACC (A) and AUC (B) scores of which the reference are set as the top 200, 400, 600, 800 and 1000 genes sorted by adjusted P values from the bulk data. (PDF) [file pcbi.1012339.s012.pdf]

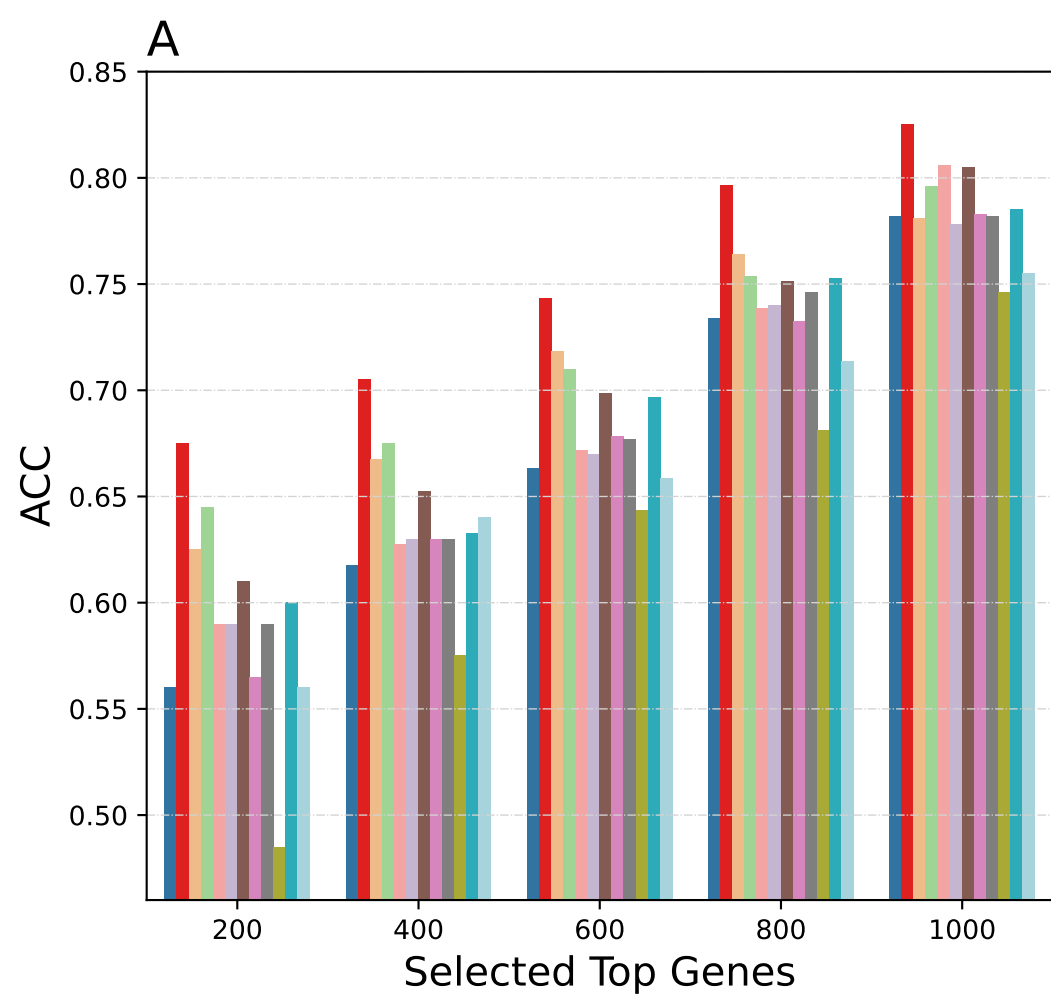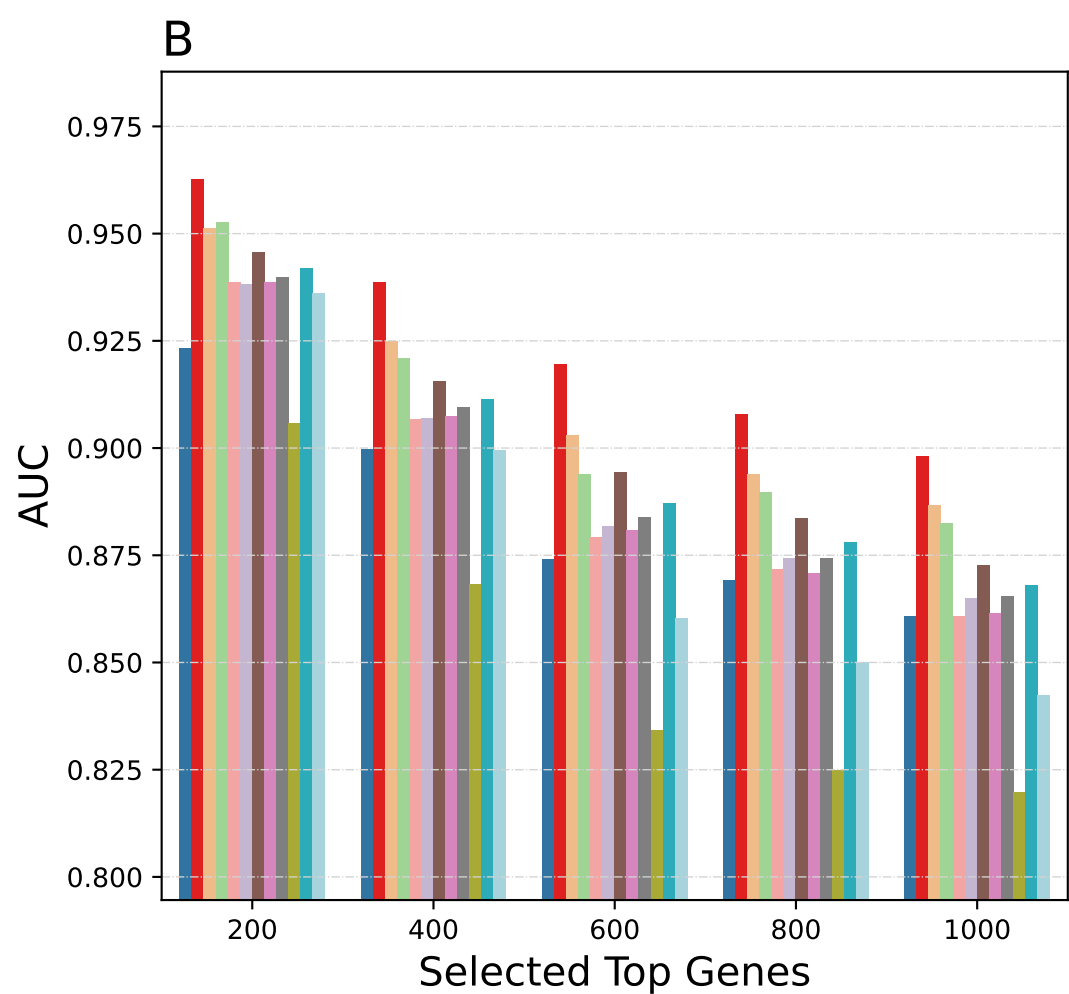

Imputation methods

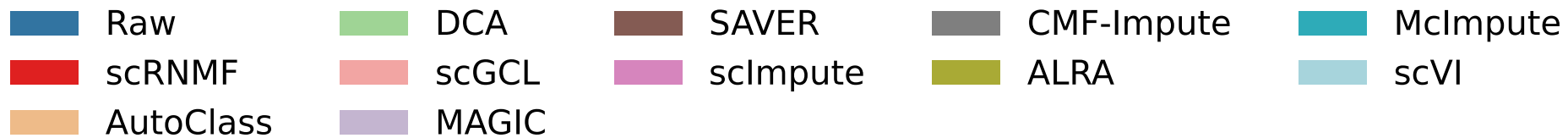

Supplement: S14 Fig — The ACC (A) and AUC (B) scores of which the reference are set as the top 200, 400, 600, 800 and 1000 genes sorted by adjusted P values from the bulk data. (PDF) [file pcbi.1012339.s015.pdf]

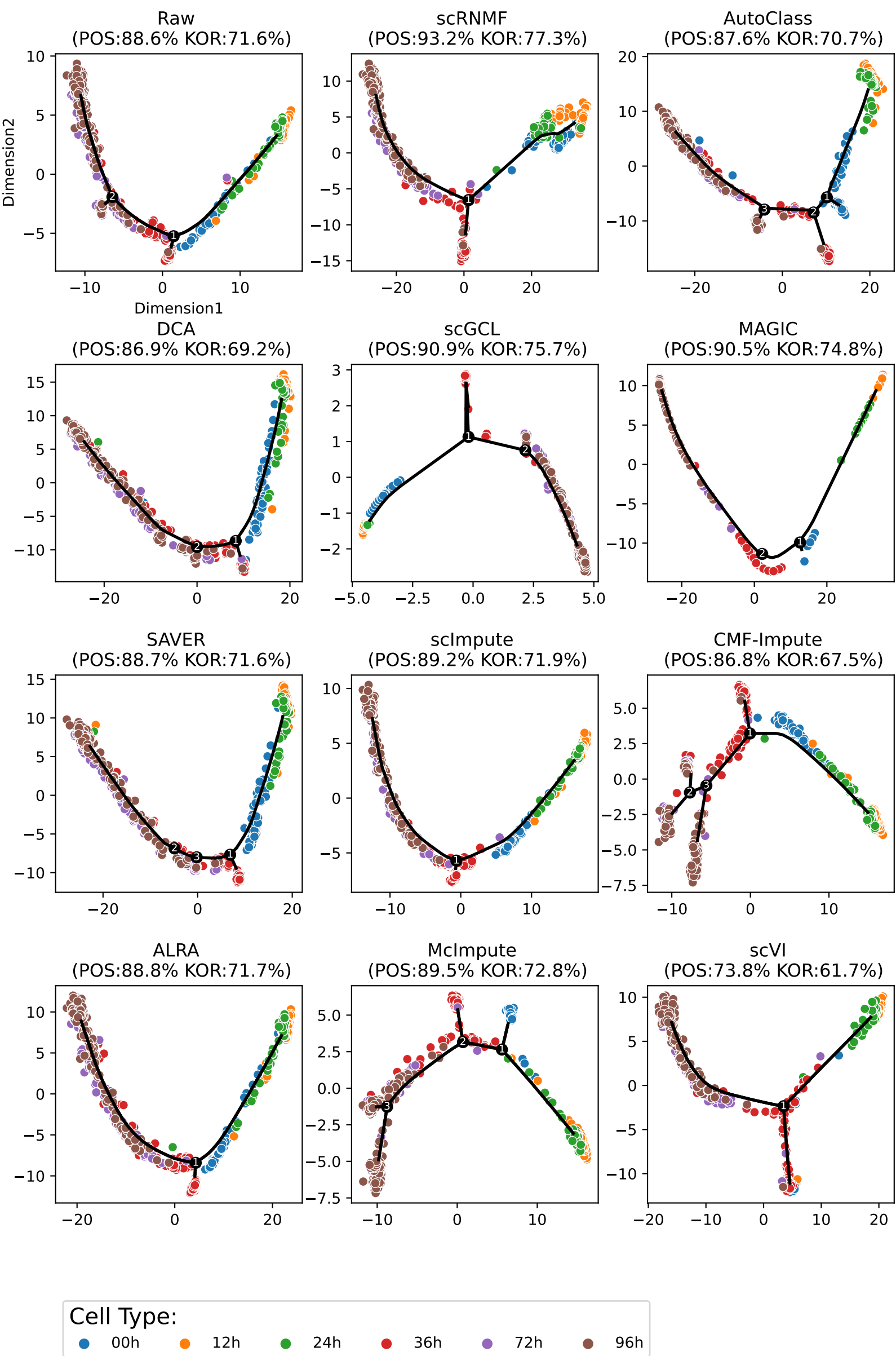

Supplement: S15 Fig — (PDF) [file pcbi.1012339.s016.pdf]
