# Supplementary material for: scRNMF: An imputation method for single-cell RNA-seq data by robust and non-negative matrix factorization
Source: PLoS Comput Biol. 2024 Aug 8;20(8):e1012339. doi: 10.1371/journal.pcbi.1012339 (PMC11338450; doi:10.1371/journal.pcbi.1012339)
Supplement: S4 Fig — (PDF) [file pcbi.1012339.s005.pdf]

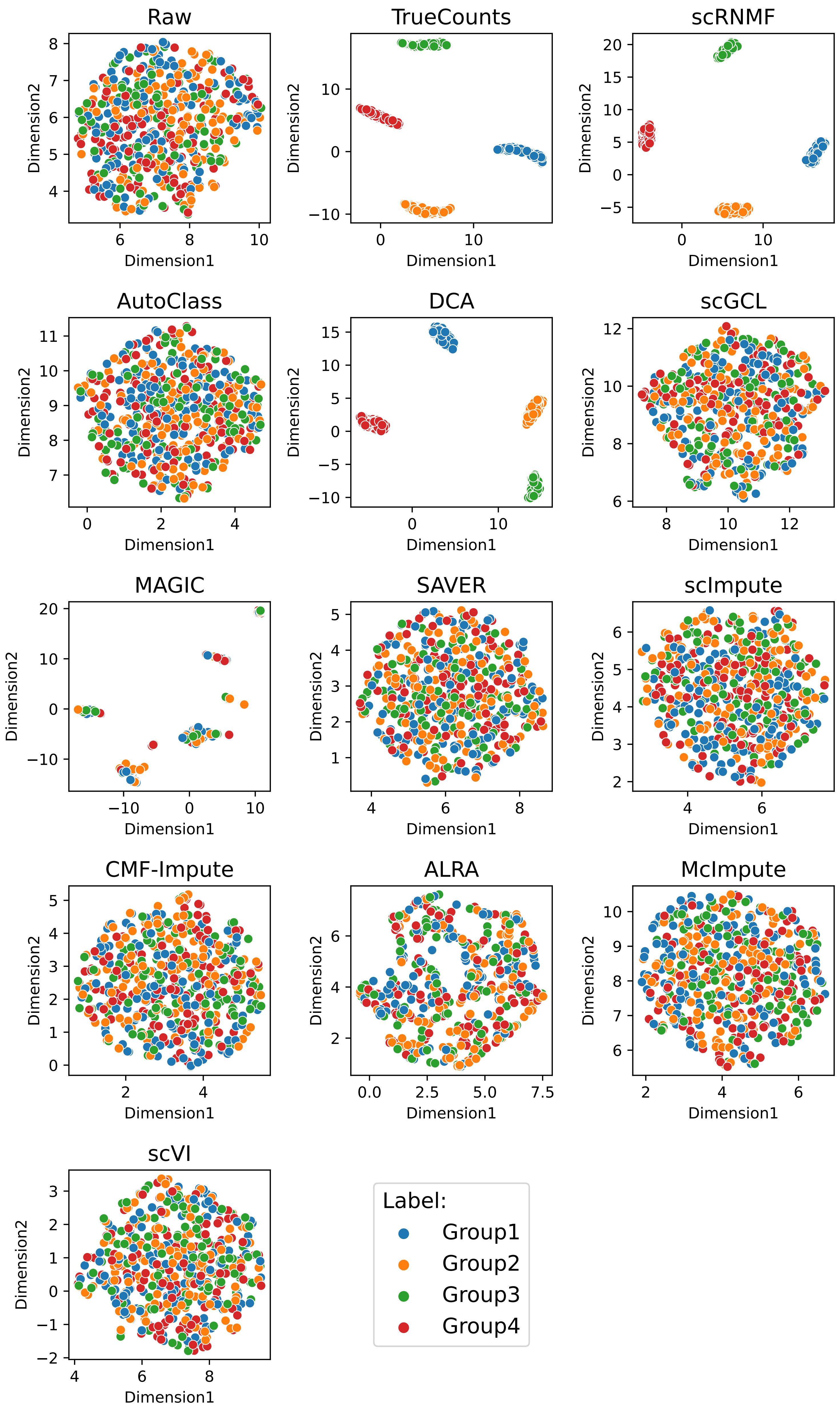
Raw
TrueCounts
scRNMF
AutoClass
DCA
scGCL
MAGIC
SAVER
scImpute
CMF-Impute
ALRA
McImpute
scVI
Dimension1
Dimension2
Label:
Group1
Group2
Group3
Group4
