# Supplementary material for: scRNMF: An imputation method for single-cell RNA-seq data by robust and non-negative matrix factorization
Source: PLoS Comput Biol. 2024 Aug 8;20(8):e1012339. doi: 10.1371/journal.pcbi.1012339 (PMC11338450; doi:10.1371/journal.pcbi.1012339)
Supplement: S13 Fig — The ACC (A) and AUC (B) scores of which the reference are set as the top 200, 400, 600, 800 and 1000 genes sorted by adjusted P values from the bulk data. (PDF) [file pcbi.1012339.s014.pdf]

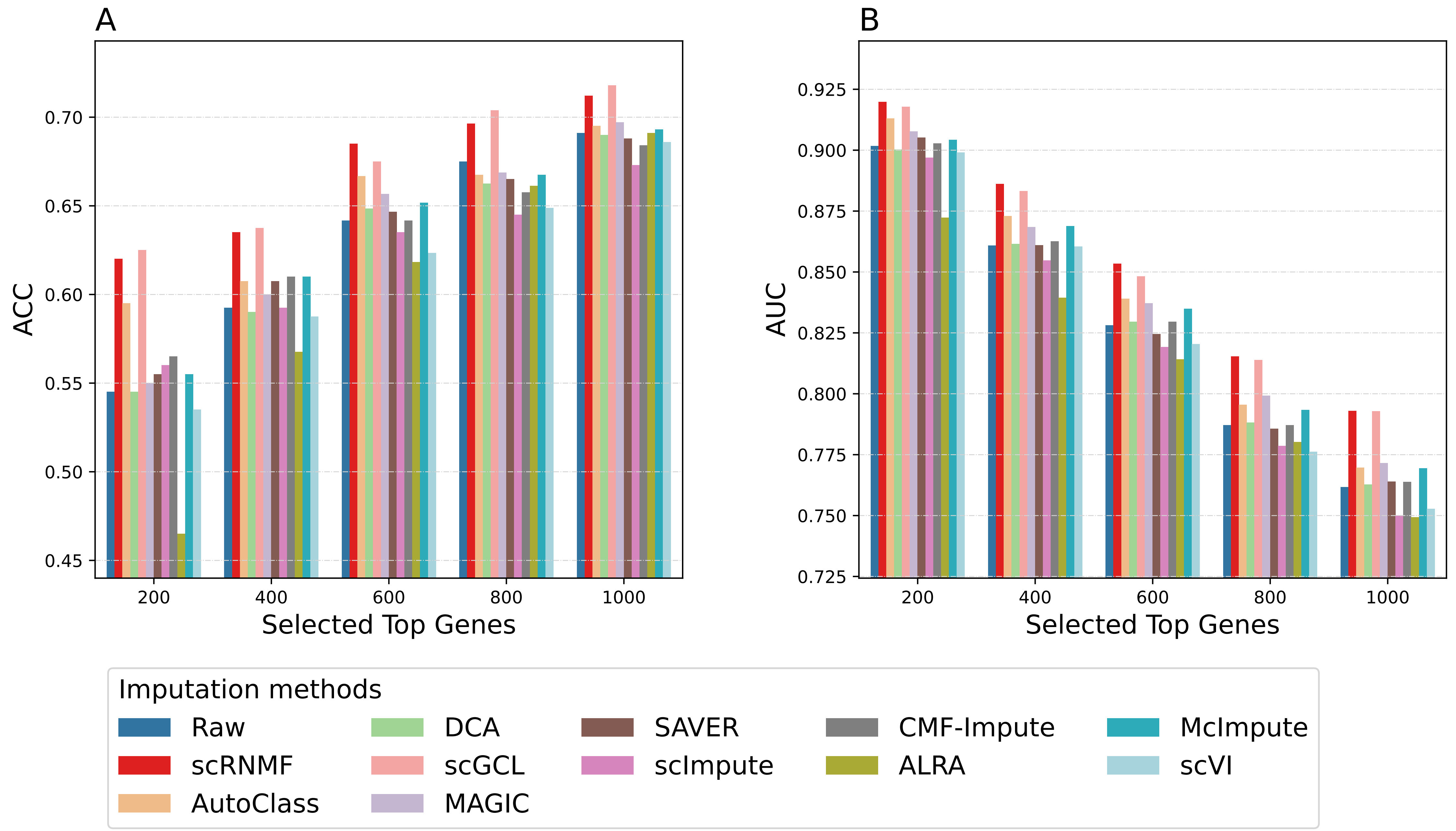

A
ACC
0.70
0.65
0.60
0.55
0.50
0.45
200
400
600
800
1000
Selected Top Genes
B
AUC
0.925
0.900
0.875
0.850
0.825
0.800
0.775
0.750
0.725
200
400
600
800
1000
Selected Top Genes
Imputation methods
Raw
scRNMF
AutoClass
DCA
scGCL
MAGIC
SAVER
scImpute
CMF-Impute
ALRA
McImpute
scVI
