# Supplementary material for: scRNMF: An imputation method for single-cell RNA-seq data by robust and non-negative matrix factorization
Source: PLoS Comput Biol. 2024 Aug 8;20(8):e1012339. doi: 10.1371/journal.pcbi.1012339 (PMC11338450; doi:10.1371/journal.pcbi.1012339)
Supplement: S16 Fig — (PDF) [file pcbi.1012339.s017.pdf]

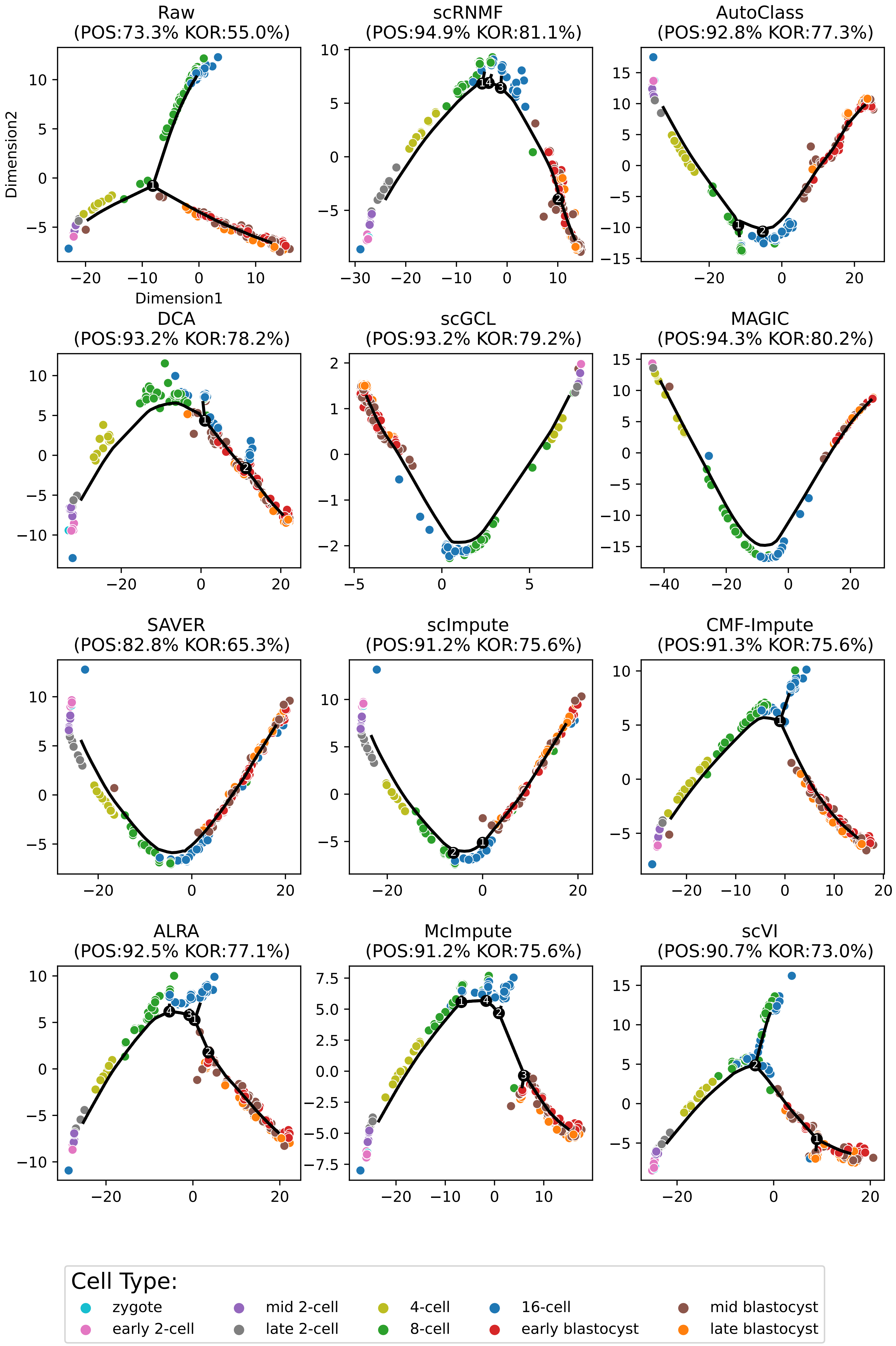
Raw
(POS:73.3% KOR:55.0%)
scRNMF
(POS:94.9% KOR:81.1%)
AutoClass
(POS:92.8% KOR:77.3%)
DCA
(POS:93.2% KOR:78.2%)
scGCL
(POS:93.2% KOR:79.2%)
MAGIC
(POS:94.3% KOR:80.2%)
SAVER
(POS:82.8% KOR:65.3%)
scImpute
(POS:91.2% KOR:75.6%)
CMF-Impute
(POS:91.3% KOR:75.6%)
ALRA
(POS:92.5% KOR:77.1%)
McImpute
(POS:91.2% KOR:75.6%)
scVI
(POS:90.7% KOR:73.0%)
Dimension1
Dimension2
Cell Type:
zygote
early 2-cell
mid 2-cell
late 2-cell
4-cell
8-cell
16-cell
early blastocyst
mid blastocyst
late blastocyst
