# Supplementary material for: scRNMF: An imputation method for single-cell RNA-seq data by robust and non-negative matrix factorization
Source: PLoS Comput Biol. 2024 Aug 8;20(8):e1012339. doi: 10.1371/journal.pcbi.1012339 (PMC11338450; doi:10.1371/journal.pcbi.1012339)
Supplement: S1 Table — (PDF) [file pcbi.1012339.s023.pdf]

CONTENTS

The parameters of all datasets.

Parameter	k	σ	α	β	λ
Simulated 1	10	10^{-2}	10^{-4}	10^{-3}	10^{-5}
Simulated 2	10	10^{-2}	10^{-4}	10^{-3}	10^{-5}
Simulated 3	10	10^{-2}	10^{-4}	10^{-3}	10^{-5}
Simulated 4	10	10^{-2}	10^{-4}	10^{-3}	10^{-5}
Simulated 5	10	10^{-2}	10^{-4}	10^{-3}	10^{-5}
Simulated 6	10	10^{-2}	10^{-4}	10^{-3}	10^{-5}
Simulated 7	10	10^{-3}	10^{-4}	10^{-3}	10^{-5}
Simulated 8	10	10^{-3}	10^{-4}	10^{-3}	10^{-5}
Simulated 9	30	10^{-3}	10^{-4}	10^{-3}	10^{-5}
Buettner	2	10^{-3}	10^{-4}	10^{-2}	10^{-5}
Usoskin	50	10^{-5}	10^{-1}	1	10
Lake	40	10^{0}	10^{-3}	10^{-5}	10^{-1}
Diaphragm	50	1	10^{-1}	10^{-1}	10^{-4}
Muscle	60	10^{-4}	10^{-1}	10^{-1}	10^{-2}
Cell Type	10	10^{-1}	10^{-1}	10	10^{-2}
Deng	30	1	10	10^{-5}	10^{-2}
Time-course	50	10^{-2}	10^{-4}	10	10^{-1}
