# Supplementary material for: scRNMF: An imputation method for single-cell RNA-seq data by robust and non-negative matrix factorization
Source: PLoS Comput Biol. 2024 Aug 8;20(8):e1012339. doi: 10.1371/journal.pcbi.1012339 (PMC11338450; doi:10.1371/journal.pcbi.1012339)
Supplement: S2 Table — (PDF) [file pcbi.1012339.s024.pdf]

CONTENTS

The details of the scRNA-seq datasets.

Dataset	Number of Cells	Number of Genes	Number of Cell Types	Zero Rate	Evaluation	Source
Simulated 1	500	1000	4	78%	Gene expression data recovery	Splatter[1]
Simulated 2	500	1000	4	71%	Gene expression data recovery	Splatter[1]
Simulated 3	500	1000	4	63%	Gene expression data recovery	Splatter[1]
Simulated 4	500	1000	4	55%	Gene expression data recovery	Splatter[1]
Simulated 5	500	1000	4	48%	Gene expression data recovery	Splatter[1]
Simulated 6	500	1000	4	42%	Gene expression data recovery	Splatter[1]
Simulated 7	500	1000	4	86%	Gene expression data recovery	Splatter[1]
Simulated 8	500	1000	4	94%	Gene expression data recovery	Splatter[1]
Simulated 9	2000	500	20	90%	Cell clustering analysis	Splatter[1]
Buettner	182	8989	3	38%	Cell clustering analysis	[2]
Usoskin	622	17772	4	96%	Cell clustering analysis	[3]
Lake	8592	34305	11	73%	Cell clustering analysis	[4]
Diaphragm	1858	22966	6	91%	Cell clustering analysis	GSM4505405
Muscle	3855	22966	6	91%	Cell clustering analysis	GSM4505405
Cell Type	1018	19097	7	50%	Gene differential expression analysis	GSE75748
Deng	286	18884	10	56%	Cellular trajectories reconstruction	[5]
Time-course	758	19189	6	55%	Cellular trajectories reconstruction	[6]

REFERENCES

1. L. Zappia, B. Phipson, and A. Oshlack, "Splatter: simulation of single-cell rna sequencing data," Genome biology **18**, 174 (2017).
2. F. Buettner, K. N. Natarajan, F. P. Casale, *et al.*, "Computational analysis of cell-to-cell heterogeneity in single-cell rna-sequencing data reveals hidden subpopulations of cells," Nat. biotechnology **33**, 155–160 (2015).
3. D. Usoskin, A. Furlan, S. Islam, *et al.*, "Unbiased classification of sensory neuron types by large-scale single-cell rna sequencing," Nat. neuroscience **18**, 145–153 (2015).
4. B. B. Lake, S. Chen, B. C. Sos, *et al.*, "Integrative single-cell analysis of transcriptional and epigenetic states in the human adult brain," Nat. biotechnology **36**, 70–80 (2018).
5. Q. Deng, D. Ramsköld, B. Reinius, and R. Sandberg, "Single-cell rna-seq reveals dynamic, random monoallelic gene expression in mammalian cells," Science **343**, 193–196 (2014).
6. L.-F. Chu, N. Leng, J. Zhang, *et al.*, "Single-cell rna-seq reveals novel regulators of human embryonic stem cell differentiation to definitive endoderm," Genome biology **17**, 1–20 (2016).
