# Supplementary material for: scRNMF: An imputation method for single-cell RNA-seq data by robust and non-negative matrix factorization
Source: PLoS Comput Biol. 2024 Aug 8;20(8):e1012339. doi: 10.1371/journal.pcbi.1012339 (PMC11338450; doi:10.1371/journal.pcbi.1012339)
Supplement: S3 Table — (PDF) [file pcbi.1012339.s025.pdf]

CONTENTS

The details of the competing imputation methods.

Methods	Year	Based approach	Available code (https://github.com/)	Reference
AutoClass	2022	Autoencoder with classifer	datapplab/AutoClass	[1]
DCA	2019	Autoencoder	theislab/dca	[2]
scGCL	2023	Graph contrastive learning	zehaoxiong123/scGCL	[3]
MAGIC	2018	Markov affinity	KrishnaswamyLab/MAGIC	[4]
SAVER	2018	Possion-Gamma model	mohuangx/SAVER	[5]
scImpute	2018	Gamma-Normal mixture model	Vivianstats/scImpute	[6]
CMF-Impute	2020	Collaborative matrix factorization	JunlinXu/CMF-Impute	[7]
ALRA	2022	Low-rank matrix approximation	KlugerLab/ALRA	[8]
McImpute	2019	Nuclear norm minimization	aanchalMongia/McImpute-scRNAseq	[9]
scVI	2018	Hierarchical bayesian model	scverse/scvi-tools	[10]

REFERENCES

1. H. Li, C. R. Brouwer, and W. Luo, "A universal deep neural network for in-depth cleaning of single-cell rna-seq data," Nat. Commun. **13**, 1901 (2022).
2. G. Eraslan, L. M. Simon, M. Mircea, *et al.*, "Single-cell rna-seq denoising using a deep count autoencoder," Nat. communications **10**, 390 (2019).
3. Z. Xiong, J. Luo, W. Shi, *et al.*, "scgcl: an imputation method for scrna-seq data based on graph contrastive learning," Bioinformatics **39**, btad098 (2023).
4. D. Van Dijk, R. Sharma, J. Nainys, *et al.*, "Recovering gene interactions from single-cell data using data diffusion," Cell **174**, 716–729 (2018).
5. M. Huang, J. Wang, E. Torre, *et al.*, "Saver: gene expression recovery for single-cell rna sequencing," Nat. methods **15**, 539–542 (2018).
6. W. V. Li and J. J. Li, "An accurate and robust imputation method scimpute for single-cell rna-seq data," Nat. communications **9**, 997 (2018).
7. J. Xu, L. Cai, B. Liao, *et al.*, "Cmf-impute: an accurate imputation tool for single-cell rna-seq data," Bioinformatics **36**, 3139–3147 (2020).
8. G. C. Linderman, J. Zhao, M. Roulis, *et al.*, "Zero-preserving imputation of single-cell rna-seq data," Nat. communications **13**, 192 (2022).
9. A. Mongia, D. Sengupta, and A. Majumdar, "Mcimpute: matrix completion based imputation for single cell rna-seq data," Front. genetics **10**, 9 (2019).
10. R. Lopez, J. Regier, M. B. Cole, *et al.*, "Deep generative modeling for single-cell transcriptomics," Nat. methods **15**, 1053–1058 (2018).
