# Supplementary material for: scRNMF: An imputation method for single-cell RNA-seq data by robust and non-negative matrix factorization
Source: PLoS Comput Biol. 2024 Aug 8;20(8):e1012339. doi: 10.1371/journal.pcbi.1012339 (PMC11338450; doi:10.1371/journal.pcbi.1012339)
Supplement: S19 Fig — (PDF) [file pcbi.1012339.s020.pdf]

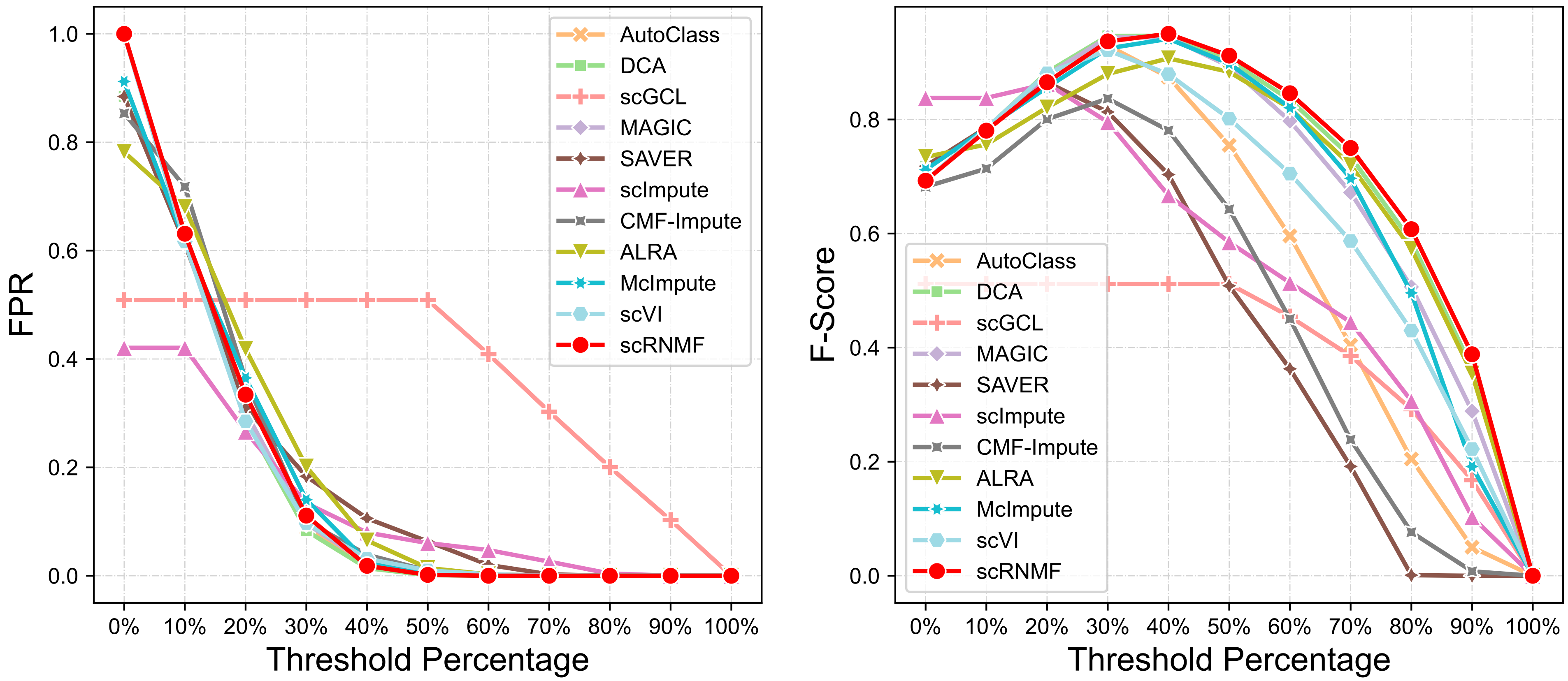

FPR
1.0
0.8
0.6
0.4
0.2
0.0
0%
10%
20%
30%
40%
50%
60%
70%
80%
90%
100%
Threshold Percentage
AutoClass
DCA
scGCL
MAGIC
SAVER
scImpute
CMF-Impute
ALRA
McImpute
scVI
scRNMF
F-Score
0.8
0.6
0.4
0.2
0.0
0%
10%
20%
30%
40%
50%
60%
70%
80%
90%
100%
Threshold Percentage
AutoClass
DCA
scGCL
MAGIC
SAVER
scImpute
CMF-Impute
ALRA
McImpute
scVI
scRNMF
