# Supplementary material for: scRNMF: An imputation method for single-cell RNA-seq data by robust and non-negative matrix factorization
Source: PLoS Comput Biol. 2024 Aug 8;20(8):e1012339. doi: 10.1371/journal.pcbi.1012339 (PMC11338450; doi:10.1371/journal.pcbi.1012339)
Supplement: S21 Fig — (PDF) [file pcbi.1012339.s022.pdf]

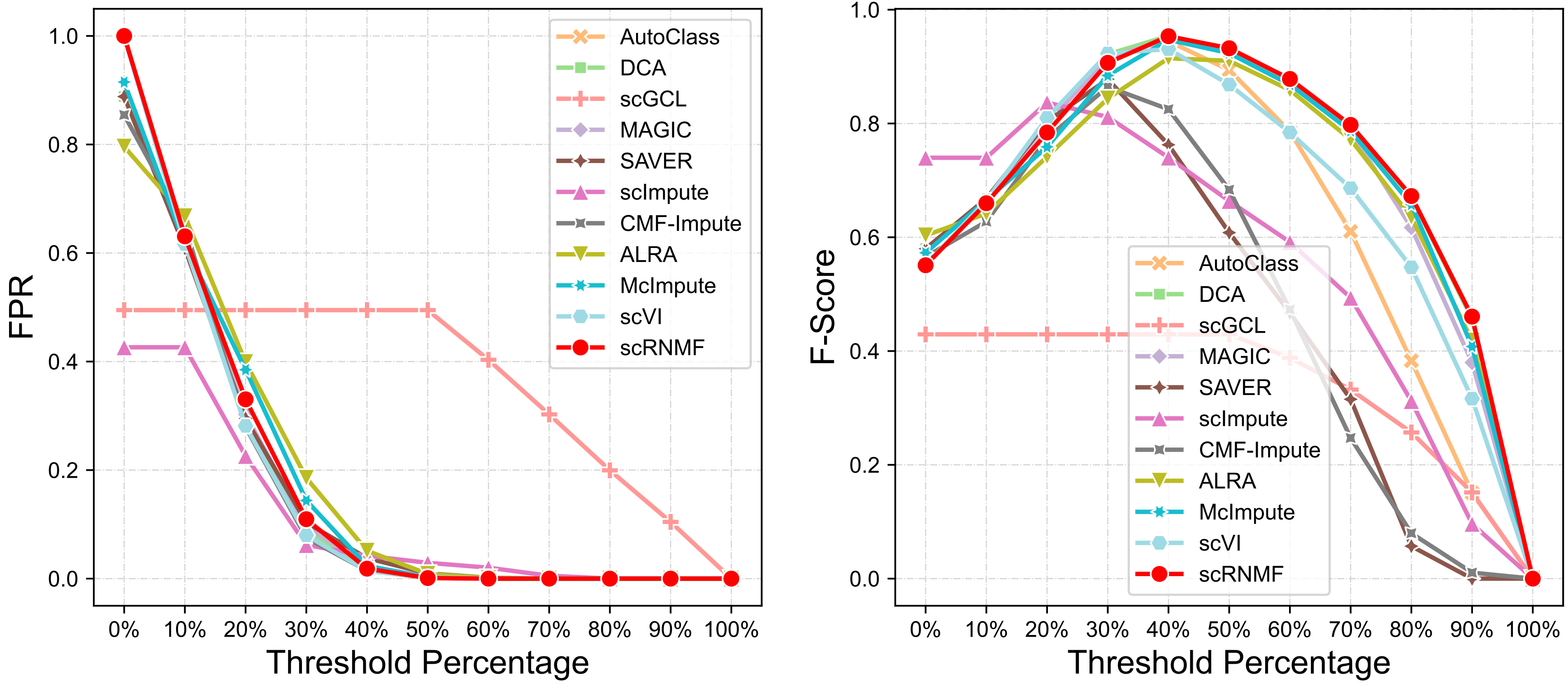
FPR
Threshold Percentage
0% 10% 20% 30% 40% 50% 60% 70% 80% 90% 100%
0.0 0.2 0.4 0.6 0.8 1.0
AutoClass
DCA
scGCL
MAGIC
SAVER
scImpute
CMF-Impute
ALRA
McImpute
scVI
scRNMF
F-Score
Threshold Percentage
0% 10% 20% 30% 40% 50% 60% 70% 80% 90% 100%
0.0 0.2 0.4 0.6 0.8 1.0
AutoClass
DCA
scGCL
MAGIC
SAVER
scImpute
CMF-Impute
ALRA
McImpute
scVI
scRNMF
